# Mechanisms mediating effects of cardiotonic steroids in mammalian blood cells

**DOI:** 10.3389/fphar.2025.1520927

**Published:** 2025-03-24

**Authors:** Yuri M. Poluektov, Olga D. Lopina, Maria A. Strelkova, Iuliia D. Kuleshova, Alexander A. Makarov, Irina Yu. Petrushanko

**Affiliations:** ^1^ Engelhardt Institute of Molecular Biology Russian Academy of Sciences, Moscow, Russia; ^2^ Faculty of Biology, Lomonosov Moscow State University, Moscow, Russia

**Keywords:** cardiotonic steroids, blood, blood cells, red blood cells, Na,K-ATPase, Src kinase, signalling cascades

## Abstract

Cardiotonic steroids (CTSs) were known as steroidal plant compounds that exert cellular effects by the binding to Na,K-ATPase. Earlier, plant (exogenous) CTSs were used to treat chronic heart failure. By now, endogenous CTS have been identified in mammals, and their concentrations in the blood, normally in a subnanomolar range, are altered in numerous pathologies. This indicates their role as endogenous regulators of physiological processes. CTS transport occurs primarily in the blood, yet the CTS effects on blood cells remain poorly understood. This review summarizes the CTS effects on blood cells of animals and humans under normal and pathological conditions, and analyzes their action based on known mechanisms of action in mammalian cells. At high concentrations (greater than 10^−9^ M), CTS binding to Na,K-ATPase inhibits the enzyme, whereas lower concentrations of CTSs induce signaling cascades or activate the enzyme. All these mechanisms are shown to be present in blood cells. The particular CTS effect is determined by the CTS type, its concentration, the isoform composition of the catalytic α-subunit of Na,K-ATPase in the cell, and other cell features. It has been demonstrated that all blood cell types (erythrocytes, leukocytes, and platelets) expressed both ubiquitously distributed α1-isoform and tissue-specific α3-subunit, which exhibits a different ion and CTS affinity compared to α1. This results in a wide spectrum of blood cell responses to fluctuations in CTS levels in the blood. In particular, an increase in the level of endogenous CTSs by a more twofold is sufficient to induce a decline in the activity of erythrocyte Na,K-ATPase. The administration of exogenous CTSs is able to modulate the proinflammatory activity of leukocytes, which is attributed to the activation of signaling cascades, and to exert an influence on platelet activation. Hence, alterations of CTS levels in bloodstream significantly affect the functionality of blood cells, contributing to the organism’s adaptive response. On top of this, a comparison of the effects of CTSs on human leukocytes and rodent leukocytes carrying the CTS-resistant α1-isoform often reveals opposite effects, thus indicating that rodents are an unsuitable model for studying CTS effects on these cells.

## 1 Introduction

Cardiotonic steroids (CTS) are a group of steroid compounds derived from certain plants and animals. CTS are selective inhibitors of Na,K-ATPase, so for a long time they have been widely used in medicine for example, to treat heart failure (On the Action of Digitalis, and Its Uses in Diseases of the Heart, 1845; Heidenreich et al., 2022). Over time, doctors gradually began to declined to prescribe this group of drugs due to their narrow therapeutic range and numerous serious adverse effects. However, a decrease of the attention to CTSs as to pharmaceutical drugs for heart failure was accompanied by the growth of interest to them as to class of specific Na,K-ATPase inhibitors (Orlov et al., 2021; Blaustein and Hamlyn, 2024). Emerging data on the effect of CTSs on signaling cascades and cell viability impelled a search for a rationale behind the existence of such a subtle biological regulator. A substantial number of studies devoted to the potential use of CTSs in the treatment of neurodegenerative, oncological, immune, and other diseases “revitalized” the already “written off” group of the drugs. By 1990s, the initial focus of investigations on exogenous (mainly plant-derived) CTSs has shifted to the role of endogenous CTSs and their regulatory effects, which became a popular trend in biology and physiology. The role of endogenous CTSs was examined in details with regard to the pathogenesis of cardiovascular diseases (Pavlovic, 2020; Orlov et al., 2021; Blaustein et al., 2022). A large number of researchers have unanimously stated that endogenous CTSs are complex regulators of the development of pathological processes. However, the exact cause-and-effect relationship that would unambiguously explain the processes of synthesis, utilization, and the role of endogenous CTSs has not yet been established. The effects of CTSs within the context of various regulatory schemes have already been described, but the majority of studies was devoted to arterial hypertension, heart and renal failure, cancer (Mijatovic et al., 2007; Mijatovic et al., 2012; Mijatovic and Kiss, 2013), aging (Guerrero et al., 2019; Triana-Martínez et al., 2019), and neuroinflammation (Orellana et al., 2016). The effects of both endo- and exogenous CTSs on different blood cells have been comparatively much less extensively investigated. This review addresses the mechanisms by which CTSs exert their effects in mammalian cells through their interaction with Na,K-АТPase, which results in its inhibition, activation and induction of signal cascades. Furthermore, the specific characteristics of CTSs actions on Na,K-ATPase isozymes containing different α-isoforms, peculiarities of action of different CTSs on Na,K-ATPase conformations, and the influence of different oxygenation level on these effects will be discussed in detail. The implementations of these mechanisms in the blood cells, including erythrocytes, leukocytes, and thrombocytes, have also been addressed.

## 2 Cardiotonic steroids. Mechanism of action

### 2.1 Sources of CTSs and their structure

СTSs were first isolated from the leaves of *Digitalis purpurea* and *Digitalis lanata*, whose extract has a long history of therapeutic use (On the Action of Digitalis, and Its Uses in Diseases of the Heart, 1845; Heidenreich et al., 2022). The medicinal use of CTSs, particularly digitalis isolated from *Digitalis purpurea*, was described as early as the 18th century (Mease, 1799). By 19th century, the use of digitalis and other plant-derived CTSs (i.e., Strophantin) for the treatment of cardiovascular diseases was already widely discussed (On the Action of Digitalis, and Its Uses in Diseases of the Heart, 1845; Fothergill, 1869; Balfour, 1895; Hatcher, 1907). In the 20th century, the study of the potential of CTSs in the therapy of these diseases continued (Howarth et al., 1947) and is still the subject of intensive research (Hollman, 1985; Fürstenwerth, 2012; Orlov et al., 2021).

**FIGURE 1:**
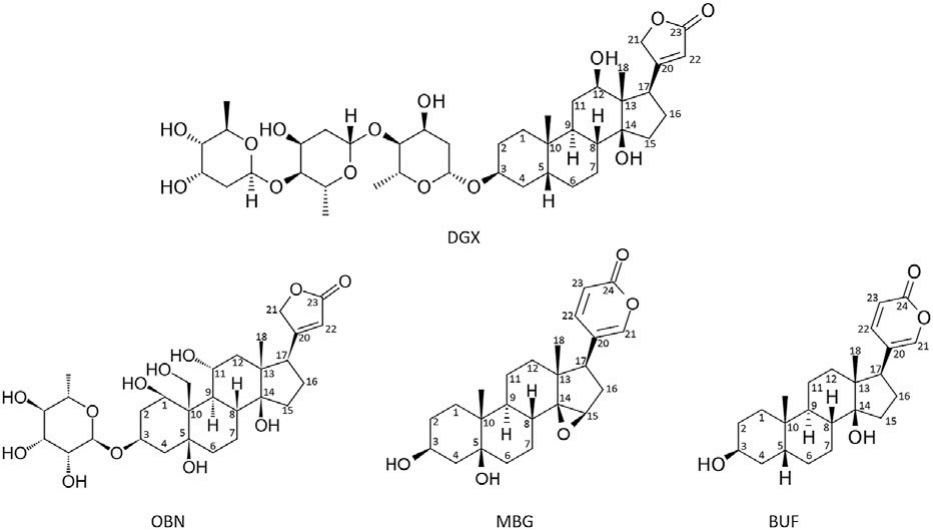
Formulas of the main cardenolides (digoxin (DGX) and ouabain (OBN)) and bufadienolides (bufalin (BUF) and marinobufagenin (MBG)). Images of CTS (2D structures) were obtained from PubChem (https://pubchem.ncbi.nlm.nih.gov/) and edited manually in MS PowerPoint.

The obtained pure compounds were used to treat heart failure (Hollman, 1985; Orlov et al., 2021). Their use in clinical practice has led to the search for other sources of CTSs (Hollman, 1985; Orlov et al., 2021). The first plаnt origin CTSs, digoxin and digitoxin, were classified as cardenolides (Hollman, 1985). Later CTSs were found in amphibians and named after the common toad (lat. *Bufa Bufa*) – bufadienolides (Lichtstein et al., 1993). Moreover, endogenous CTSs have also been found in the mammalians (Hollman, 1985; Kieval et al., 1988; Hamlyn et al., 1991; Orlov et al., 2021). A characteristic feature of CTSs is the presence of a steroid core, a lactone ring at position C-17 and a hydroxyl group at C-14. CTSs are divided into two classes: cardenolides and bufadienolides. Cardenolides have a five-membered ring at C17, while bufadienolides have a six-membered lactone ring at C17 with two double bonds (Mijatovic et al., 2007). Besides, СTSs may also contain sugar residues at C3 position (ouabain, digoxine), in contrast with aglicones (marinobufagenin, bufalin). Figure 1 shows the structures of cardenolides - digoxin and ouabain and bufadienolides - bufalin and marinobufagenin.

### 2.2 Na,K-ATPase as a target of CTS and current view on the mechanisms involved in CTS effects

Currently, the only receptor known to bind CTSs is Na,K-ATPase. Na,K-ATPase is an ATP-dependent transmembrane ion transporter of potassium and sodium ions (Na-pump), that was found in all types of animal cells (Kaplan, 2002). The main function of Na,K-ATPase is to maintain ionic homeostasis of the cell. Na,K-ATPase is composed of two main subunits: α and β. Complexes of α- and β-subunits form functional dimer (αβ)2. The α-subunit of Na,K-ATPase (∼110 kDa) has 10 transmembrane segments (H1-H10). The CTS binding site as well as the binding sites for ATP, sodium and potassium ions are located on the α-subunit. The α-subunit participates in ATP hydrolysis through conformational changes of the enzyme, switching the enzyme between E1 and E2 conformations that switch the enzyme affinity to Na^+^ and K^+^, respectively. Additionally, its transient phosphorylation at the aspartate residue in the active site, associated with the EP conformation of the enzyme, promotes the electrogenic exchange of 3 intracellular sodium ions for 2 extracellular potassium ions.

Mammals express four α- (α1, α2, α3, α4), and three β-isoforms (β1, β2, β3), encoded by different genes. The α1-subunit is present in all mammalian cells. Three other α-isoforms are tissue-specific. The α2 is found in astrocytes, cardiomyocytes, skeletal muscle, and glial cells, and α3-isoform in neuronal cells, heart (Clausen et al., 2017). The α4-subunit was firstly found in mammalian testes. The β-subunit is a glycoprotein (protein part 35 kDa), represented by 3 isoforms, is required for the transport of newly synthesized Na,K-ATPase into the membrane, as well as for the correct localization of the α-subunit in the cytoplasmic membrane and for K^+^-dependent reaction of the enzyme (Chow and Forte, 1995). The extracellular domain of the β-subunit contributes to determining the kinetics of K^+^ interaction (Chow and Forte, 1995). Tilt angle of transmembrane helix of β-subunit affect the interaction between β-subunit and the C-terminus of α-subunit and thereby the relative stability of the E1P and E2P states (Hilbers et al., 2016). As result β2 in contrast to β1 and β3 more effectively stabilizes occluded E1P state relative to the outward-open E2P state of enzyme that leads to the decrease of K^+^ affinity (Hilbers et al., 2016). The γ-subunit of Na,K-ATPase (FXYD family homolog), which contributes to the regulation of enzymatic activity, was firstly found in kidney cells, and later 6 different isoforms of γ-subunit were identified (Meyer et al., 2020). The following subchapters examine the features of the interaction of СTSs with various isoforms of Na,K-ATPase and the cellular effects that this interaction caused by various conditions.

#### 2.2.1 High doses of CTSs inhibit Na,K-ATPase, whereas enhancing its activity and inducing signaling cascades at low doses

The therapeutic effect of CTSs in the treatment of cardiovascular failure has been suggested to be the result of the inhibition of the transport function of Na,K-ATPase. Disruption of Na,K-ATPase functionality leads to impaired ion transport, in particular, to intracellular Na^+^ load and K^+^ depletion, which, in turn, affect membrane potential, Na^+^/Ca^2+^ exchanger activation and accumulation of the intracellular calcium. Therefore, the inhibition of myocardial Na,K-ATPase results in the augmented calcium level in cardiomyocytes and, consequently, to the increased myocardial contractility (Pavlovic, 2020). Moreover, these alterations in ion homeostasis may impair cell adhesion and intercellular interactions (Cereijido et al., 2012; Vagin et al., 2012; Larre et al., 2014). The β-subunit of Na,K-ATPase, functioning as an adhesion molecule, is apparently involved in this process (Vagin et al., 2006). Na,K-ATPase molecules located at the cell-cell junction act as adhesion molecules, interacting with each other via β1-subunits (Tokhtaeva et al., 2011; Vagin et al., 2012), while the cytosolic domain of the α1-subunit interacts with E-cadherin and the ankyrin/spectrin cytoskeleton (James Nelsont and Veshnock, 1987; Zhang et al., 1998; Vagin et al., 2012). At low concentrations (<100 nM), ouabain increases the hermeticity of the epithelial cell tight junction, whereas at higher concentrations (>300 nM), on contrary, decreases it (Larre et al., 2010; Larre et al., 2014). The role of the β1-subunit of Na,K-ATPase in cell adhesion was also demonstrated in the chinese hamster ovary cells (CHO-line). (Vilchis-Nestor et al., 2019). It was shown that nanomolar concentrations (50 nM) of ouabain, which do not inhibit Na,K-ATPase increase adhesion between CHO cells expressing β1.

The long-term practice of CTSs use for the treatment of chronic heart failure has been associated with a number of adverse effects, such as myocardial hypertrophy, which may be a consequence of pathologically activated signaling cascades (Liu et al., 2016).

Importantly, while high doses of CTSs inhibit Na,K-ATPase, CTSs at concentrations below IC50 are able to increase its activity (Blaustein and Hamlyn, 2024). While the inhibitory effect of ouabain, digoxin, and marinobufagenin was exerted in rat hippocampal slice culture at 1,200 nM, 100 nM, and 1,000 pM, respectively, administred at concentrations lower than inhibiting concentrations (120 nM for ouabain, 10 nM for digoxin, and 100 pM for marinobufagenin, respectively) these CTSs activated its transport activity (Oselkin et al., 2010). Moreover, ouabain at 120 nM has been shown to protect neuronal slice cell culture from hypoxia-hypoglycemia due to its stimulatory effect (Oselkin et al., 2010). Preventing the increase in Na,K-ATPase activity by 2 nM of marinobufagenin offset the protective effect of CTSs (Oselkin et al., 2010). It is known that ischemia leads to the disruption of the Na^+^ and K^+^ gradient, leading to cell death (Doyle et al., 2008). The authors of the study (Oselkin et al., 2010) suggest that the neuroprotective effect of ouabain is due to the fact that activation of Na,K-ATPase prevents the loss of the gradient of monovalent cations or promotes its more rapid restoration. Additionally, we also propose an alternative explanation based on the fact that binding to non-inhibitory concentrations of ouabain prevents glutathionylation of Na,K-ATPase (Poluektov et al., 2019), which is induced by hypoxia and causes the observed decrease of the enzyme activity (Petrushanko et al., 2012). Furthermore, the activation of Na,K-ATPase was observed at 1–10 nM ouabain on a purified Na,K-ATPase in microsomal preparation from pig kidney, which contains only α1-isoform (Tverskoi et al., 2016). Consequently, Na,K-ATPase activation also occurs in a cell-free system, although the precise mechanism of this effect remains to be elucidated. A possible mechanism of activation associated with the displacement of endogenous CTSs from the isolated Na,K-ATPase during titration of protein preparations with exogenous CTSs still remains to be proven (Blaustein and Hamlyn, 2024).

The CTS-dependent activation of signaling cascades is attributable to the multifaceted function of Na,K-ATPase, which, in addition to its role in ion transport, possesses receptor functionality that is activated by the interaction with CTSs. While inhibition of Na,K-ATPase is provided by CTS binding to the vast number of enzyme molecules, activation of signaling cascades occurs even when CTS bind an insignificant percent of molecules. Accordingly, the activation of signaling cascades can be observed at concentrations much lower than Kd (Figure 3) (Orlov et al., 2021). In turn, the CTS binding constants to Na,K-ATPase vary between different CTSs and depend on isoform type of Na,K-ATPase and its conformation (Klimanova et al., 2015; Tverskoi et al., 2021). For example, Kd values for ouabain, digoxin and marinobufagenin binding to Na,K-ATPase from pig kidney in E2P conformation are equal to 53 nM, 208 nM and 2,320 nM correspondingly (Tverskoi et al., 2021). Moreover, the comparison of Na,K-ATPase structures in the E2P conformation with and without various CTSs revealed no significant differences, suggesting that CTSs bind to a preformed cavity in Na,K-ATPase. Taken together, it implies that the activation of signaling cascades may occur as a result of fixation of the enzyme in a certain conformation upon binding of CTS, and that binding of various CTS, in particular ouabain, captures the enzyme in the E2P-like conformation (Laursen et al., 2013; 2015; Kanai et al., 2021). CTS binding to this enzyme conformation activates signaling cascades. This process is mostly mediated by the altered affinity to its protein partners, namely, Src kinase (Xie et al., 2013; Klimanova et al., 2015; El-Mallakh et al., 2019). The interaction of CTSs with Na,K-ATPase occurs on the extracellular surface of the membrane in a narrow “channel” formed by transmembrane segments of the α-subunit H1-H2, H5-H6, and H7-H8 (Lingrel et al., 1998; Tverskoi et al., 2021). The depth of the binding site localized in the channel is different for different CTS types. For instance, the binding site of marinobufagenin is 5Å closer to the channel exit than that of ouabain (Klimanova et al., 2015; Tverskoi et al., 2021). These data explain the fact that marinobufagenin exhibits equivalent binding affinity for all conformations of Na,K-ATPase with equal affinity, while ouabain displays high binding affinity (higher than that of marinobufagenin) exclusively for the E2P conformation (Klimanova et al., 2015). In addition, binding of these CTSs induces distinct conformational changes (Klimanova et al., 2015) that may result in the activation of various signaling cascades due to their interaction with its partner proteins. These data reveal potential causes of the observed differences in the effects (Akimova et al., 2005) of marinobufagenin and ouabain on cells. Additionally, *in vivo* ouabain and marinobufagenin may also modulate each other’s actions and work in synergy when are added simultaneously (Chakraborty et al., 2017). These effects may be explained by the different dependence of the binding constant on the Na,K-ATPase conformation for ouabain and marinobufagenin described above and the ability of ouabain to displace marinobufagenin from the Na,K-ATPase complex in E2P conformation (Klimanova et al., 2015).

Under hypoxic conditions, the effect of CTSs on cells is altered (Lakunina et al., 2017; Petrushanko et al., 2017). Specifically, treatment of mouse fibroblast (SC1 cell line) and human embryonic kidney cells (HEK293 cell line) expressing wild type of murine α1-subunit (Petrushanko et al., 2017) by ouabain (250 µM for 30 min) in hypoxia (0.05% pO2) in contrast to normoxia (20% pO2) does not induce the activation of Src kinase, but impairs its activating phosphorylation, typically increased for both type of cells under hypoxia. Thus, ouabain counteracts the increase in the level of the activated form of Src kinase induced by low oxygen content. This concentration of ouabain does not induce cell death during 24 h in normoxia and, moreover, ouabain have been demonstrated to enhance mouse fibroblast cell viability in hypoxic conditions (Lakunina et al., 2017). As previously mentioned, the protective effect of ouabain against hypoxia and ischemia has also been demonstrated in the rat hippocampal slice (Oselkin et al., 2010). The role of CTSs as potential antihypoxants remains to be determined. However, the increasing endogenous CTS levels observed in congestive heart failure and acute myocardial infarction (Bagrov et al., 1998; Orlov et al., 2021) may serve as indirect evidence of their antihypoxic effect. Besides, the important role of eCTSs in adaptation to hypoxia is indicated by data on increased levels of eCTS in healthy human model of acute hypoxia (divers) and diving animals (Manfrini et al., 2022). Elevated eCTSs levels were also observed in human model of chronic hypoxia - idiopathic pulmonary arterial hypertension patients (Manfrini et al., 2023). According to the data obtained, high levels of eCTSs are predictive of better adaptation of the right ventricular afterload. We believe that the antihypoxic effect is exerted by low doses of CTS, which do not cause significant inhibition of Na,K-ATPase (Oselkin et al., 2010), but are capable activate signaling cascades. High doses of CTS, leading to inhibition of Na,K-ATPase, cause sodium accumulation that, in turn, increase calcium level and myocardial contractility. It is possible that this could improve tissue blood supply and temporarily reduce the effect of hypoxia on the entire body. However, the question of the effect of large therapeutic concentration exogenous CTS on the heart under hypoxic conditions remains open. The reasons for the frequent problems of digoxin therapy in patients with chronic pulmonary failure were discussed. For example, in an isolated heart model, it was shown that pre-hypoxia reduces the contractility-enhancing effect of digoxin (Allam et al., 1990).

Thus, when considering the action of CTSs, it is necessary to take into account their concentrations and type of cells and to remember the possibility of inhibitory and activating action, as well as activation of signaling cascades.

#### 2.2.2 Sensitivity of Na,K-ATPase isozymes with different alpha subunit isoforms to CTSs

The affinity of Na,K-ATPase for CTS depends on its subunit composition. In 1987, it was discovered that canine cardiac myocytes contain two isoforms of Na,K-ATPase α-subunit, which differ in molecular weight and affinity to ouabain: the dissociation constants are 2 nM and about 300 nM for high and low affinity isoforms, correspondently (Maixent et al., 1987). At that time, it was also suggested that the toxicity of CTSs, which were used as drugs, was related to their inhibition of the low-affinity Na,K-isoform, which is now known as the Na,K-ATPase containing α1-subunit. The subsequent research revealed that the presumed high-affinity isoform is actually comprised of two isoforms, now known as Na,K-ATPase containing α2 and α3 (Blanco and Mercer, 1998). The α4-subunit, which was discovered later, also exhibits low affinity for ouabain (Blanco et al., 1999) (Table 1).

**TABLE 1:** Properties of different isoforms of the catalytic alpha subunit of Na,K-ATPase.

Isoforms	Ouabain Sensitivity (Mobasheri et al., 2000; Rodrigues-Mascarenhas et al., 2009)	K^+^ K _0.5_ mM (Crambert et al., 2000; Larsen et al., 2014)	Na^+^ K _0.5_ mM (Zahler et al., 1997; Blanco and Mercer, 1998; Crambert et al., 2000)	Oxidation Sensitivity (Xie et al., 1995; Segall et al., 2003; Bogdanova et al., 2006; Petrushanko et al., 2012; Xianyu et al., 2014)
α1	low	1	10	
				low
α2		3	10	high
	Intermediate (Rodrigues-Mascarenhas et al., 2009)/high (Mobasheri et al., 2000)			
α3		1	25–50	high
	high			
α4		2.14 ± 1.14^a^ (Woo et al., 1999)	9.13 ± 1.81^a^ (Woo et al., 1999)	
	Low (300 нМ Кd) (Woo et al., 1999) K_i_ 1.6 ± 1 × 10^–9^ M (Wagoner et al., 2005)	5.9 ± 1.1 (Blanco et al., 1999)	13.5 ± 1.3 (Blanco et al., 1999)	

^a^ Na+ inhibition of [3 H^+^] ouabain binding and K+ inhibition of [3 H^+^] ouabain binding.

Further studies have shown that the sensitivity of different isoforms of Na,K-ATPase to CTS depends also on CTS type and its sugar content. According to the data on binding affinity of CTS containing sugar moieties (glycosides, which includes also ouabain), digitalis glycosides exhibit significant selectivity (up to 4-fold) for human α2- and α3-subunits over α1-, in contrast to aglycones (bufadienolides, namely, bufalin and marinobufagenin), which bind α1-, α2- and α3-isoforms with indistinguishable dissociation constants (Katz et al., 2010). Binding constants for ouabain vary across animal species (for rev see (Lingrel, 2010)). The Kd values for all studied mammalian α-subunit isoforms, excluding those of rodents, vary in the nanomolar range. All human isoforms are highly affine to ouabain (Crambert et al., 2000; Wang et al., 2001), but α2β isozyme have higher association and dissociation rate constants for ouabain than other isozymes. Additionally, potassium ions exert a more pronounced effect on the ouabain affinity for the α1β isozyme than for α2β and α3β. Potassium cations located outside the cell prevent ouabain binding because of E2P dephosphorylation, so it causes enzyme transition to E2·2K^+^, to which CTSs cannot readily bind (Kanai et al., 2020). K^+^/ouabain antagonism has been shown to protect α1-subunit from the CTS-induced inhibition within physiological potassium concentrations (Crambert et al., 2000). This effect is the result of the substitution of amino acid residues within the M1-M2 extracellular loop of the α2-subunit and M7-M8 hairpin (Crambert et al., 2004). Сooperation of amino acid residues at position 119 and 124 within the M1-M2 with M7-M8 hairpin is responsible for isoform-specific differences in ouabain binding kinetics (Crambert et al., 2004). Moreover, K^+^/ouabain antagonism depends on the structure of the lactone ring of CTSs, hydrogen bonds and steric constraints of CTS (Kanai et al., 2020). CTSs with five-membered lactones (cardenolides), differ significantly in their affinity to Na,K-ATPase in the presence of potassium than six-membered ones (bufadienolides) (Azalim et al., 2020). In all, the manifestations of potassium/ouabain antagonism for α-subunit isoforms will be expressed differently for various CTS.

**FIGURE 2:**
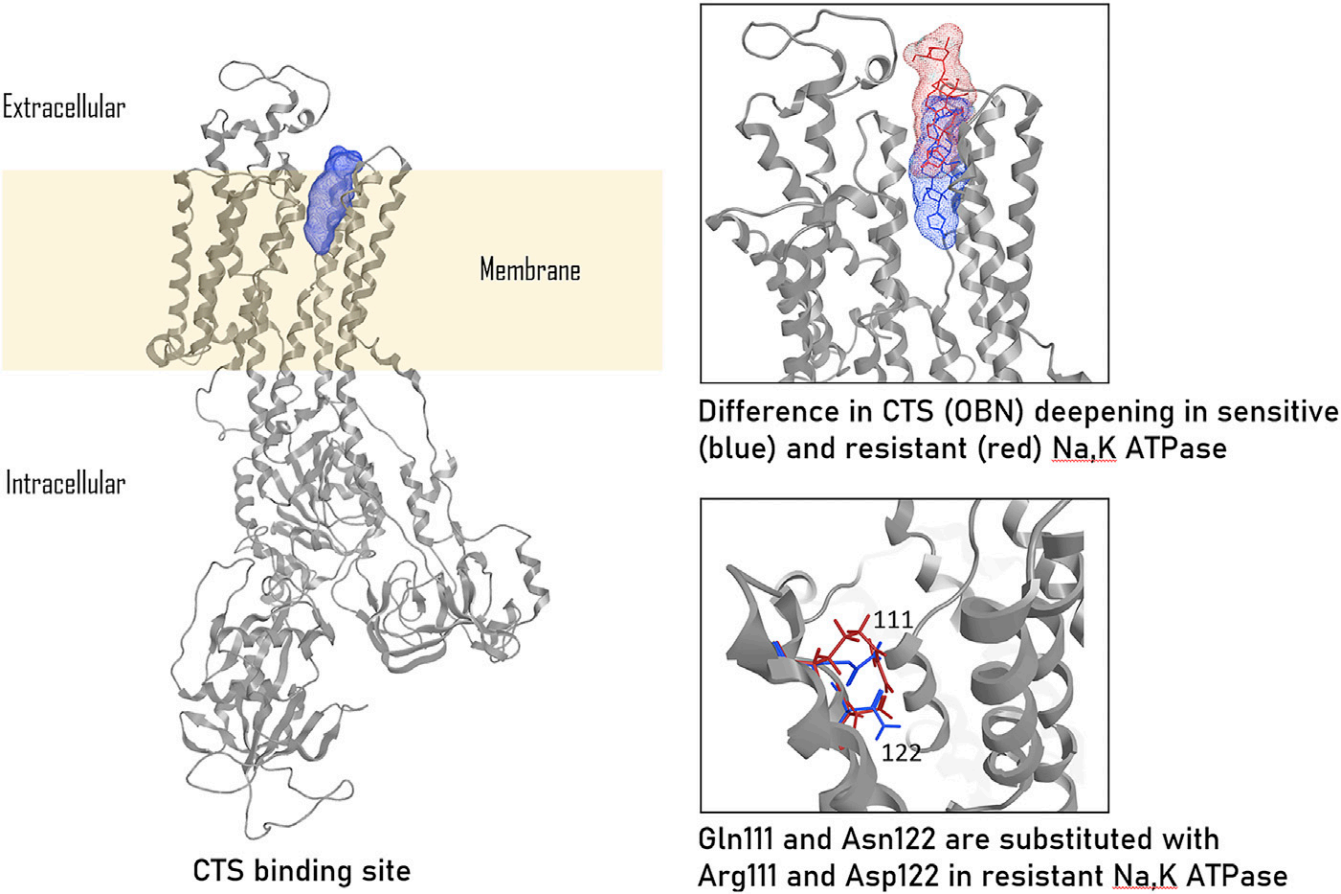
CTS binding to ouabain-sensitive and ouabain-resistant Na,K-ATPase, according to (Tverskoi et al., 2021). The ouabain (OBN) binding localization in sensitive Na,K-ATPase is colored in blue, ouabain binding localization in resistant (rat) Na,K-ATPase is colored in red. The presented images were based on the models published by us earlier (Tverskoi et al., 2021) and created in Moe2013.10.

The reasons underlying the different sensitivity of isoforms to СTSs have been undergone the most extensive study in rodents, which exhibit the disparity in CTS affinity of three orders of magnitude comparing to Na,K-ATPase from other species. To be more precise, the α1-subunit of rodent Na,K-ATPase, also known as CTS-resistant α1R-Na,K-ATPase, shows a ∼1000-fold lower affinity for ouabain compared to α2, α3-subunits of rodents and α1-subunit of other mammalian species (Lingrel et al., 1998). Rat Na,K-ATPase that contains the α1-isoform binds ouabain with Kd of 3,226 nM (Tverskoi et al., 2021), whereas Kd values for rat Na,K-ATPase with α2-, α3-, and α4- isoforms are 115 nM, 1.6 nM, and 312 nM, respectively (O’Brien et al., 1994a; Woo et al., 1999). Data on the inhibition of Na,K-ATPase activity shows that the rat α1-isoform has an IC50 of 48,000 nM, whereas the IC50 of the rat α2- and α3-isoforms are 58 nM and 6.7 nM, corespondigly (O’Brien et al., 1994b). The impaired binding of the rat α1-isoform to CTSs is caused by the replacement of uncharged amino acids Gln111 and Asn122 with charged Arg and Asp (Price and Lingrel, 1988; Lingrel, 2010; Tverskoi et al., 2021) (Figure 2). This amino acid substitution dramatically reduces the ouabain affinity of the α-subunit-Na,K-ATPase as result of decrease in the depth of CTS entry into the binding channel (Figure 2) (Tverskoi et al., 2021). Thus, for rat Na,K-ATPase expressed in Sf-9 insect cells, the value of dissociation constant for ouabain are 4.3 ± 1.9 × 10^−5^ M for rat α1β1; 1.7 ± 0.1 × 10^−7^ M for α2β1; and 3.1 ± 0.3 × 10^−9^ M for α3β1 (Blanco and Mercer, 1998). Despite the presence of an ouabain-resistant isoform, physiological concentrations of ouabain in rodents are approximately equivalent to those observed in humans (0.34 ± 0.06 nM vs. 0.152–0.53 ± 0.10 nM) (Orlov et al., 2021), implying the presence of alternative ways of CTS-dependent regulatory mechanisms in rodents. The expression of the CTS α1-resistant isoform, rather than downstream cascades, is responsible for the resistance of rodent cells to CTSs (Akimova et al., 2015). In cell lines, ouabain treatment for 24 h in the concentration range of 100 nM-10 µM induced cell death in human α1-sensitive Na,K-ATPase-expressing cells, while no such effect was observed in murine α1-resistant Na,K-ATPase-expressing cells, even at ouabain concentrations of up to 3 mM (Akimova et al., 2015).

In addition to their divergent affinities for CTSs, the discovered isoforms exhibit different affinities for sodium and potassium ions. It is hypothesized that the different affinities of the Na,K-ATPase isoforms for its ligands are closely related to their physiological functions. The data obtained by (Mobasheri et al., 2000) demonstrates a higher affinity of tissue-specific α2- and α3-isoforms for ouabain compared to the ubiquitous α1-isoform. The α2-isoform colocalizes with Na^+^/Ca^2+^ exchanger (Juhaszova and Blaustein, 1997), participating in the regulation of calcium levels in muscle cells. Affinity for potassium depends on both α- and β–isoforms. Particularly, α2- in complex with β1-subunit shows slightly lower afinity for potassuim than α1- and α3-isoforms with β1-subunit (Crambert et al., 2000). In combination with β2-subunit, however, it exhibits a significantly reduced affinity for potassium compared to other isoforms (K_0.5_ = 3 mM compared to K_0.5_ ∼ 1 mM for other isoforms) (Crambert et al., 2000; Larsen et al., 2014) (Table 1). In glia and astrocytes, the α2β2 Na,K-ATPase is supposed to be especially effective in facilitating potassium influx after intense neuronal activity. The α3-subunit, which is widely represented in neurons, is optimized to stimulate the efflux of sodium ions that follows neuronal excitation (Azarias et al., 2013). This isoform is therefore characterized by a lower Na^+^ sodium affinity with a K_0.5_ of 25–50 mM, in comparison to other isoforms (for which K_0.5_ is approximately 10 mM) (Zahler et al., 1997; Blanco and Mercer, 1998) (Table 1). It should be taken into account that β-subunit is also able to influence ion binding and transport, which is supported by the fact that the K^+^ half-activation constant (K_1/2_) of the α1β3 Na,K-ATPase was higher than that of the α1β1 Na,K-ATPase isozymes in the presence of external Na^+^(Jaisser et al., 1994).

It should also be noted that CTS-sensitive tissue-specific α2- and α3-isoforms exhibit heightened sensitivity to oxidation and glutathionylation (Petrushanko et al., 2012; Xianyu et al., 2014) than the α1-isoform (Table 1). However, this phenomenon is not associated with the number of cysteine residues in the isoform. According to PubMed NCBI sequence library, the number of cysteine residues for α1- and α2-subunits of Norway rat is equal, whereas α3-subunit insignificantly exceeds this number. While the number of cysteines remains consistent between various α-isoforms, they are differently arranged in the tertiary structure of the α-subunit (Bogdanova et al., 2006). Thus, the oxidation and glutathionylation sensitivity of α-isoforms is determined by the structural arrangement of cysteine residues and, particularly, by the shift in E2-E1 structural equilibrium of the α2-subunit towards the E1 conformation, which is more open to the cytosol (Segall et al., 2003; Bogdanova et al., 2006; Poluektov et al., 2019). The properties of different isoforms are summarized in Table 1.

In all, depending on the proportion of α-subunit isoforms, each cell exhibits different sensitivity to CTS and oxidative stress.

#### 2.2.3 Endogenous mammalian CTSs

Both cardenolides and bufadienolides are present in the human body. First, the endogenous ouabain (cardenolide) was detected in blood plasma (Ludens et al., 1991). Later, endogenous marinobufagenin (bufadienolide) was detected in plasma and urine (Ludens et al., 1991; Bagrov et al., 1998; Fedorova et al., 1998). Currently, ouabain (Schneider et al., 1998; Kawamura et al., 1999), digoxin (Goto et al., 1990), bufalin (Lichtstein et al., 1993), marinobufotoxin (Yoshika et al., 2007), marinobufagenin (Bagrov and Fedorova, 1998; Fedorova et al., 1998), telocinobufagin (Haddy and Overbeck, 1976; Hamlyn et al., 1991; Komiyama et al., 2005; Orlov et al., 2021); and several digoxin-immunoreactive compounds (Weinberg et al., 1992) have been found in human plasma and urine (Goto et al., 1988; Goto et al., 1990). Goto et al. described the elevated urinary excretion of digitalis-like factor under salt-loading conditions, underlining its involvement in regulation of hemostasis (Goto et al., 1989). The range of CTS concentrations in blood varies from subnanomolar to nanomolar (Orlov et al., 2021). The most studied are endogenous marinobufagenin and ouabain. Both of them are vasoconstrictors (Bagrov et al., 1998) that are involved in the cardiac contraction, heart rate modulation, blood pressure regulation and natriuresis, and play an important role in the pathogenesis of salt-sensitive hypertension (for rev see (Pavlovic, 2020; Orlov et al., 2021; Blaustein et al., 2022). Based on the studies (Fedorova et al., 2005a; Strauss et al., 2020), one can suggest that increased salt intake causes an increase in the pituitary endogenous ouabain, which, in turn, stimulates the renin–angiotensin–aldosterone system and sympathetic activity, triggering the synthesis of marinobufagenin in the adrenal glands (Słabiak-Błaż and Piecha, 2021). Their plasma concentrations vary in the subnanomolar range but do not exceed 1 nM (Gottlieb et al., 1992; Lewis et al., 1994; Rossi et al., 1995; Bagrov et al., 1998; Gonick et al., 1998; Lopatin et al., 1999; Wang et al., 2003), yet pathological concentrations can be up to several nM (Orlov et al., 2021), in particular, ouabain level significantly increase under essential hypertension, primary aldosteronism (Rossi et al., 1995), preeclampsia (Lopatin et al., 1999). Marinobufagenin levels are increased in the patients with primary aldosteronism, essential hypertension, congestive heart failure (Gonick et al., 1998). preeclampsia (Lopatin et al., 1999), rats with high salt diet (Fedorova et al., 2002; 2005b). Notably, urinary excretion is increased by 38 times under high-salt diet in comparision low-sat diet of CTSs (1.8 pmol/per day) and by 50 times under intravenous saline infusion (from 0.1 to 5 pmol/per hour) (Goto et al., 1989). Marinobufagenin and ouabain effectively inhibit Na,K-ATPase containing ouabain-sensitive α-isoform at concentrations of 2.0 and 0.8 µM, respectively (Tverskoi et al., 2021). It is assumed that the physiological effects of endogenous CTSs (eCTS), whose concentrations lie in the subnanomolar (norm) or nanomolar range (pathology), is mediated probably through the activation of signaling cascades in particular via Src kinase associated to Na,K-ATPase or by inhibition of small fraction of Na,K-ATPase (Tverskoi et al., 2016).

Synthesis of eCTSs was shown to take place in the adrenal glands. However, adrenalectomy does not result in a complete loss of eCTSs in the blood (Schoner and Scheiner-Bobis, 2007). This fact implies the presence of other organs and tissues involved in the biosynthesis of eCTSs. Ouabain has been detected in the cerebrospinal fluid of mammals, including humans, at nanomolar concentrations. The level of ouabain in human CSF (∼2 nM) was 4 times higher compared to its concentration in blood serum (Dvela et al., 2012). Recent data on tissue distribution of ouabain after intraperitoneal ouabain injection in mice showed that injected ouabain is not detected in the brain, indicating that it does not cross the blood-brain barrier (Abaimov et al., 2024). Together with the above data, this also indirectly indicates independent synthesis of ouabain in the brain. According to Weidemann et al. (Weidemann et al., 2004) and Manunta et al. (Manunta et al., 2006) the tissue content of CTSs remains stable in hypothalamus, but plasma levels decreased in adrenal-deficient animals, thus indicating that brain is not sufficient source of plasma CTS. These findings indirectly indicate that brain CTS are unable to cross blood-brain barrier in normal conditions. Digoxin, which is more hydrophobic than ouabain, has been shown to penetrate to the brain (Taskar et al., 2017), being associated with a number of side effects on the central nervous system (El-Mallakh et al., 1995). In the study of Taskar (Taskar et al., 2017), digoxin showed low brain-to-plasma concentration ratio ∼0.07 when administered at 2 mg/kg, intravenously in rats. Inhibition of efflux transporter P-glycoprotein (P-gp) by elacridar resulted in 12-fold increase in brain-to-plasma ratio. These finding highlights the importance of membrane transporters in CTS transition in or out the brain. Endogenous ouabain is currently considered not only as a circulating hormone acting in the CNS, but also a paracrine neurohormone produced locally in CNS (probably by the hypothalamus) (Weidemann et al., 2004; Leenen et al., 2017). A greater increase in the level of CTSs in the hypothalamus of athymic nude mice than in control mice after intraperitoneal administration of salt and the absence of such effects in the plasma and in the adrenal glands indicate independent CTS synthesis in the hypothalamus (Weidemann et al., 2004). By now, the studies of Takahasi (Takahasi et al., 1988; Leenen et al., 1993) on the functioning and synthesis of CTS in the brain (hypothalamus) have been substantiated (Kawamura et al., 1999; Gomez-Sanchez et al., 2010; Taves et al., 2011; Hamlyn et al., 2014).

A heightened levels of eCTSs has been observed in response to various stimuli, including hypoxia (De Angelis and Haupert Jr, 1998) and coincides with the development of several pathologies, namely, myocardial (Bagrov et al., 1998) and renal ischemia (Tian et al., 2010), hypertension (Schoner and Scheiner-Bobis, 2007), sepsis (Raimondo et al., 2023) and other, increasing the CTS levels from two- to threefold fold (for rev. see (Blaustein and Hamlyn, 2020; Orlov et al., 2021)). The accelerated rise in CTS in response to stressful stimuli, such as cardiac surgery (Simonini et al., 2015) or conditions associated with salt excess or deficiency (Manunta et al., 2006), or increasing CTS level in the hypothalamus of athymic nude mice under intraperitoneal saline injection (Weidemann et al., 2004) also allow us to consider them as stress hormones (Weidemann et al., 2004; Blaustein and Hamlyn, 2020).

#### 2.2.4 Signaling cascades

Src kinase has been shown to form a complex with α1-containing Na,K-ATPase, and this interaction is highly specific. Accepted model of signal transduction in this complex implies two interaction interfaces: the SH2 domain of Src kinase binds to the actuator domain of the α1-subunit of Na,K-ATPase, while its kinase domain binds to the nucleotide-binding domain, preventing Src kinase from autophosphorylation and activation. When ouabain binds to the extracellular domain of Na,K-ATPase, the interaction between the kinase domain of Src and the nucleotide-binding domain of the pump is disrupted. The kinase domain is released, leading to its autophosphorylation and activation of the kinase. At the same time, the interaction between the SH2 domain of Src and the actuator domain of Na,K-ATPase is preserved (Li and Xie, 2009; Banerjee et al., 2015). Src can transmit the activating signal to mitogen-activated kinases, Erk1/2-kinase, induce further cascades via phospholipase C or the PI3-kinase- mediated signaling pathway (Reinhard et al., 2013).

For the long time, it has been assumed that only α1-isoform is involved in interaction with Src kinase, since it has been the only isoform known to contain the predicted Src interaction interface (Li and Xie, 2009; Karpova et al., 2010; Petrushanko et al., 2017). However, recent evidence suggests that the α2-subunit may also be involved in interaction with Src, and its knockout leads to impaired signaling transmission via CTSs (Rognant et al., 2022). It is assumed that two “populations” of Na,K-ATPase in cells are distinguished: free, or pumping, Na,K-ATPase, which mainly performs transport activity and accounts for about 30% of the total amount of Na,K-ATPase on the cell surface, and non-pumping Na,K-ATPase which includes Src-associated Na,K-ATPase (Liang et al., 2007). CTS binding to non-pumping Na,K-ATPase leads to Src-dependent transactivation of receptor tyrosine kinases (RTKs) such as the EGF receptor (EGFR), which converts CTS binding to activation of serine/threonine kinases, lipid kinases and lipases (Chen et al., 2018). CTS binding has been shown to induce the endocytosis of Na,K-ATPase via the Src-dependent pathway (Liu et al., 2004) or Src-independent pathway (Cherniavsky-Lev et al., 2014). Src activation is independent of sodium, potassium and calcium ions concentrations and leads to the changes in the expression of more than 100 genes, demonstrating a fourfold increase in c-Fos mRNA within 30 min after ouabain addition (Taurin et al., 2002).

Direct interaction of Src kinase and Na,K-ATPase has been shown many times, both *in vitro* and on cell cultures (Tian et al., 2006; Banerjee et al., 2015; Nie et al., 2020; Petrushanko et al., 2022), and the dissociation constant of this complex was recently measured (Kd = 0.21 ± 0.04 µM) (Petrushanko et al., 2022). All these data makes reliable the mechanism of signal transduction through direct interaction between Src kinase and Na,K-ATPase.

Src kinase is the most studied Na,K-ATPase protein partner, but not the only one. In Caco-2 human colorectal adenocarcinoma cells, about 10 proteins are shown to be associated with Na,K-ATPase after treatment by ouabain (Akimova et al., 2016). Some of them are able to affect the transport activity of Na,K-ATPase, similarly to CTSs: for example, the homolog of the γ-subunit of Na,K-ATPase phospholemman, or agrin, which interacts with α3-subunit of Na,K-ATPase. An important protein partner of Na,K-ATPase is caveolin 1 (Wang et al., 2004; Zhang et al., 2020), the major protein of caveolae, the bulb-shaped membrane invaginations that play an important role in membrane transport, response to external signals and are linked to numerous signaling pathways (for review see (Parton et al., 2018)). The α1-subunit of Na,K-ATPase is shown to interact with caveolin 1 by NH2-terminal cytosolic tail (Cai et al., 2008). The caveolin-1 protein together with Src mediates cholesterol-dependent (Zhang et al., 2020) and CTS-dependent (Liu, 2005) regulation of Na,K-ATPase isoformal ratio at the surface of cells, inducing the endocytosis of Na,K-ATPase together with its signaling protein partners.

In mammalian cells, the receptor function of Na,K-ATPase activated by CTSs also include Src-independent pathways. Namely, ouabain binding to Na,K-ATPase in neurons changes the sensitivity of NMDA receptors, which are colocalized with Na,K-ATPase in cholesterine-rich membrane rafts. In subnanomolar concentrations, ouabain inhibits NMDA-receptors (Sibarov et al., 2023), whereas its nanomolar concentrations cause their hypersensitization, which does not involve Src kinase activation (Akkuratov et al., 2020). Besides, low ouabain concentrations lead to the activation of Erk1/2-kinase but not Src kinase in cells that contain α3-subunit of Na,K-ATPase (Karpova et al., 2010; Madan et al., 2017). In fibroblasts, PI3K1A and Akt kinases are also activated in the presence of ouabain via Src-independent pathway (Wu et al., 2013). All these observations imply the existence of other possible proteins that can mediate CTS-dependent signaling pathways.

The role of CTSs in the pathogenesis of various diseases has been reviewed in detail (Orlov et al., 2021). In short, the highest concentrations of endogenous ouabain are observed in essential hypertension, while the increased levels of both ouabain and marinobufagenin are observed in primary hyperaldosteronism; besides, marinobufagenin is elevated in chronic renal failure. Taking into account the complex development of the pathological patterns in these diseases, the role of CTSs in every particular disease requires further study. Thus, it would be beneficial to undertake further investigations of the effect of CTSs on different types of cells, organs and tissues. Given that blood cells are continuously exposed to circulating endogenous CTSs, as well as to the therapeutic doses of exogenous CTSs, the aim of our review is to systematize and analyze the data on the impact of diverse CTSs on various blood cells and to elucidate potential molecular mechanisms underlying these effects.

## 3 Effect of CTS on blood cells

Blood consists of blood plazma and various types of blood cells. CTS effect on the whole blood is basically determined by their effect on each type of blood cells. In a number of pathological conditions (heart failure, chronic renal failure, hypertension, etc.), the concentration of endogenous CTSs in the blood is increased (Orlov et al., 2021), exerting various regulatory effects on certain components of the circulatory system. The study of systemic effects in the absence of connection with pathological conditions is a complex and nearly impossible task; therefore, we decided to focus on the individual groups of blood cells and the effects of CTSs on these cells. The vast majority of blood cells (more than 99% of all cells) is a fraction of erythrocytes, which constitutes about 40% of all blood volume. Leukocytes and thrombocytes compose about 1% of the blood volume (Bain, 2021). Recently, several reviews have been published, which consider immunomodulating effects of CTSs to be associated with their effects on various diseases (Schneider et al., 2017; Škubník et al., 2021; Bryniarski et al., 2022). We will describe effects of different CTSs on blood cells, namely, erythrocytes, leukocytes and thrombocytes, based on the already discovered mechanisms of CTS effects on mammalian cells.

In general, the effect of СTSs on blood cells is due to the inhibition or, at low concentrations, activation of Na,K-ATPase, and induction of signaling cascades. These effects are directly dependent on the concentration of CTS and the specific isoform, which it interacts with. Therefore, to understand the effects on cells, it is important to investigate, which isoforms are present in these cells.

### 3.1 Distribution of Na,K-ATPase isoforms in blood cells

As discussed above, the isoforms of the catalytic α-subunit of Na,K-ATPase differ in their affinity for CTSs, Na^+^, K^+^ and sensitivity to oxidative modifications, therefore the isoformal composition of Na,K-ATPase in cells largely determines their response to CTSs. Table 2 contains the generalized data on the presence of α-subunit isoforms, as well as their mRNA, in various blood cells. In leukocytes and reticulocytes, the red blood cell precursors, mRNAs of α1- and α3-subunits was detected (Stengelin and Hoffman, 1997). It was later shown that mature red blood cells contain α1- and α3-, but not α2-isoform (Hoffman et al., 2002; Balzan et al., 2007). The same was shown for CHRF-288, a human megakaryocytic cell line, which are the precursors of platelets. The authors also claimed this for platelets themselves, where the α_3_-isoform content was significantly lower than that of the α_1_-isoform, although data were not shown (Kimura et al., 2005).

**TABLE 2:** Distribution of Na,K-ATPase catalytic α-subunit isoforms in the blood cells.

Cells/Alpha subunit	α1	α2	α3	α4
Red blood cells	+++ (Hoffman et al., 2002; Balzan et al., 2007)	- (Hoffman et al., 2002; Balzan et al., 2007) (DB1)	++ (Hoffman et al., 2002; Balzan et al., 2007)	- (DB1)
Leukocytes	++ (Stengelin and Hoffman, 1997)	+- (Stengelin and Hoffman, 1997) (DB1)	++ (Stengelin and Hoffman, 1997)	+? (DB1)
*Mononucleacytes/macrophages*	++ (DB1, DB2)	++ (DB1; DB2)	++ (DB1; DB2)	+ (DB1; DB2)
*Granular leukocytes*	+ (DB1; DB3)	+ (DB1; DB3)	+ (DB1; DB3)	- (DB1)
Lymphocytes	++ (DB1; DB3)	+ (DB1; DB3)	++ (DB1; DB3)	+ (DB1; DB3)
*NK-cells*	+ (DB1; DB2)	+ (DB1; DB2)	+ (DB1; DB2)	+ (DB1; DB2)
*CD4 Cells*	++ (DB1; DB2)	+ (DB1; DB2)	+ (DB1; DB2)	+ (DB1; DB2)
*CD8 Cells*	++ (DB1; DB2)	+ (DB1; DB2)	+ (DB1; DB2)	+ (DB1; DB2)
*T-reg*	+? (DB1)	+? (DB1)	+? (DB1)	+? (DB1)
Platelets	+++ Kimura et al. (200)	- Kimura et al. (200) (DB2)	++ Kimura et al. (200)	- (DB2; DB3)

Designations: - - no mRNA, or protein was detected. +- - negligible isosyme mRNA, content can be detected, isozyme not detected. +? - mRNA, detected, no detected isozyme reported. + - negligible isosyme content may be detected. ++ - both mRNA, and protein detected. +++ - predominant isoform. DB1 – Database: proteinatlas.org, mRNA, data (Uhlén et al., 2015). DB2 – Database: pharos.nih.gov, mRNA, and protein expression data mainly reproduced from Human Proteome Map (Kim et al., 2014). DB3 – Database: tissues.jensenlab.org, protein expression prediction.

Regarding β-isoforms, it should be noted that erythroid progenitor cells and mature erythrocytes contain β1-, β2- and β3-isoforms of Na,K-ATPase (Hoffman et al., 2002), although it was previously shown that mRNA of only β2- and β3-isoforms was found in reticulocytes (Stengelin and Hoffman, 1997). At the same time, the presence of mRNA of β1-, but not β2-subunit, was shown in leukocytes (Stengelin and Hoffman, 1997). However, it was later shown that leukocytes also express β3-subunit (Chiampanichayakul et al., 2006).

In all, the presence of α1 and α3-isoforms of Na,K-ATPase has been demonstrated for erythrocytes and platelets, and mRNA of these isoforms has been detected in leukocytes. Since the affinity of α3 for some CTS types is higher than that of α1, it can be assumed blood cells are able to respond to physiological and pathological changes in the СTS concentrations in blood. The effects of varying concentrations of CTSs on different types of blood cells are discussed below.

### 3.2 CTS effects on blood immune cells and some immune diseases

The role of CTSs in autoimmune diseases (rheumatoid arthritis, Chagrin syndrome, etc.), general inflammatory status and immune status of tumor diseases has been widely described (Kondělková et al., 2010; Huang et al., 2020). In short, CTSs mainly act as anti-inflammatory and immunosuppressive agents. Their effects on different types of immune blood cells are summarized below.

#### 3.2.1 Leukocytes

##### 3.2.1.1 Mononuclear cells/macrophages

The effect of CTSs on macrophages and monocytes (precursors of macrophages and dendritic cells) is currently being actively investigated, although the precise mechanism of their action in this type of cells has not been fully understood.

Initially, we will consider the effect of CTSs on murine macrophages, given that mice are the most common model for studying the CTS effects on macrophages. To start with, it has been demonstrated that CTSs impede macrophage migration and cytokine production in murine models. The administration of ouabain at a dose of 0.56 mg/kg to mice infected with *Leishmania* (L.) *amazonensis* resulted in a decrease in peritoneal migration of macrophages and in the production of two proinflammatory cytokines, interferon gamma (IFN-γ) and tumor necrosis factor alpha (TNF-α). At the same time, no cytotoxic effects of this ouabain dose was observed on macrophages (Jacob et al., 2013), suggesting that it does not inhibit Na,K-ATPase. The intraperitoneal injection of the equivalent doses (0.56 mg/kg) of marinobufagenin into mice decreased zymosan-activated production of proinflammatory cytokines, IL-1β and IL-6, without affecting the level of TNF-α (Carvalho et al., 2019). Experiments with zymosan-stimulated peritoneal macrophages (*in vitro*) demonstrate that lowest concentration (about 10 nM) of marinobufagenin diminished the zymozan-induced expression of IL-1β, IL-6, and TNF-α (Carvalho et al., 2019). The *in vitro* treatment of peritoneal macrophages with varying concentrations (10, 100, 1,000, and 10000 nM) of marinobufagenin demonstrated no observed cytotoxic effect (Carvalho et al., 2019). The absence of toxicity is presumably due to the murine Na,K-ATPase that contains CTS-resistant α1-isoform. Moreover, marinobufagenin exhibits 70-fold lower potency in inhibiting α2/α3-isoforms compared to ouabain (IC50 = 3.8 ± 0.9 μM vs. IC50 = 55 ± 9 nM for ouabain) and does not inhibit mice α1-isoform (for ouabain IC50 = 25 ± 12 μM) (Carvalho et al., 2019), which explains the possible differences observed in the discussed studies. The possible causes of different effects of ouabain and marinobufagenin are discussed above in Section 2. In all, both ouabain and marinobufagenin have been shown to curtail cytokine production, and since this occurs even at low concentrations that do not inhibit Na,K-ATPase, it can be assumed that the observed effects are mediated by the activation of signaling cascades.

Indeed, the action of CTSs on macrophages may be mediated by Src kinase activation. Particularly, 24 h treatment of murine peritoneal macrophages with telocinobufagin (CTS, bufanolide; 10 and 100 nM) was shown to induce an oxidative burst and enhanced NF-kB activation (Khalaf et al., 2019). Since NF-kB, an inflammatory master regulator in macrophages, (for rev. see (Mussbacher et al., 2023)) increases ROS, its activation means that pro-inflammatory activation of macrophages occurs (Forman and Torres, 2002; Mussbacher et al., 2023). Conversely, these effects were not observed after macrophages were pretreated with 1 μM pNaKtide, the specific peptide inhibitor of Na,K-ATPase-associated Src signaling, (Khalaf et al., 2019), 1 μM Src kinase inhibitor PP2, or when macrophages were isolated from Na,K-ATPase α1-subunit heterozygous null mice (Na,K-ATPase α−1+/−) compared to macrophages isolated from wild type mice (Khalaf et al., 2019). Moreover, even at a concentration of 100 nM, telocinobufagin does not affect the accumulation of sodium in cells, i.e., it does not inhibit Na,K-ATPase. These data demonstrate that telocinobufagin effect is mediated by the activation of Src kinase associated with α1-subunit of Na,K-АТPase (Khalaf et al., 2019). The mechanism of Src kinase effect on NF-kB is not shown in this study (Khalaf et al., 2019) and may be different, as follows from the various studies. In this regard, it should be noted that activation of the Src/EGFR/NF-κB signaling pathway can lead to the activation of microglia (resident macrophages of the brain) (Ma et al., 2024). Thus, Na,K-ATPase α1-Src signaling complex acts as a central component of CTS mediated pro-inflammatory response.

Resibufogenin (CTS, bufanolide) has also been shown to induce signaling pathways and to involve NF-kB in the reduction of cytokine levels. Intraperitoneal administration of resibufogenin 10 mg/kg and 20 mg/kg into mice with endotoxemia decreases TNF-α, IL-6 and monocyte chemoattractant protein 1 (MCP-1) levels in blood serum. In lipopolysaccharide (LPS)-stimulated macrophages, resibufogenin treatment at concentrations of 20, 40, 80 μM decreases the secretion of proinflammatory mediators (namely, inducible NO synthase, IL-6, MCP-1) through the suppression of their transcription in a dose-dependent manner. These effects are mediated by the suppression of IkBα phosphorylation that is necessary for the release of NF-kB from the inhibitor and its further transportation in the nucleus (Gao et al., 2022). Consequently, signaling cascades have been identified as being involved in the action of CTSs on murine macrophages.

However, when transitioning from the mouse model to human cells, an inversion of the effects of CTSs on macrophages and monocytes is observed. In human cells, CTSs do not cause suppression, but rather stimulation of cytokine production. An *in vitro* study (Teixeira and Rumjanek, 2014) showed that human monocytes treated with ouabain at a concentration of 100 nM produced heightened levels of early markers of monocyte activation: IL-1β, TNF-α, IL-10, vascular endothelial growth factor (VEGF), surface monocyte activation markers such as CD69, a transmembrane type II C-lectin receptor, and HLA-DR (Human Leukocyte Antigen–DR isotype) (Ziegler et al., 1994). Besides, it also stimulated the expression of CD80 and CD86 (B7-1 and B7-2 type I membrane protein in the immunoglobulin superfamily, correspondingly), which are considered as markers of increased cytokine expression, costimulating signals for activation and survival of T-lymphocytes (Koukourakis et al., 2023). In another study, the authors concluded that ouabain exhibits immunomodulatory effect through affecting monocyte activity (Teixeira and Rumjanek, 2014). In the study of Foey et al. (Foey et al., 1997) it was also shown that treatment of human mononuclear cells by ouabain at a concentration of 100 nM induces TNF-α and, in particular, IL-1β production, whereas IL-6 synthesis was suppressed. This concentration does not exert cytotoxic effect for human mononuclear cells during 24 h. The papers (Raviprakash and Manna, 2014; Teixeira and Rumjanek, 2014) report on the increasing immunoreactivity of human monocytes after their exposure to CTSs. A short-term exposure (within 1 h) to oleandrin (200 ng/mL, corresponding to 347 nM) enhances the biological response of human monocytes to IL-8 and increases the number of receptors for IL-8 (Raviprakash and Manna, 2014) without inducing cytotoxicity. It is important to denote that ouabain (100 nM) induces IL-1β, IL-6, and TNF-α in human peripheral blood mononuclear cells, but, when these cells were stimulated with LPS, ouabain suppressed the production of IL-6 and TNF-α. Moreover, ouabain (1 mg/kg) decrease LPS-induced lethal toxicity in mice and circulating IL-6 and TNF-α levels (Matsumori et al., 1997).

The contradictory effects of CTSs on mice and humans can be explained by different concentrations used for exposure and by different sensitivity of α1-subunits of human and mice α-isoforms of Na,K-ATPase. For instance, in study (Teixeira and Rumjanek, 2014), in which the activating effect of CTS was observed, human monocytes were treated with 100 nM of ouabain *in vitro*, whereas the inhibitory effect was described *in vivo* on mice with ouabain concentrations reaching 0.56 mg/kg (Jacob et al., 2013), when the maximum concentration in the blood is in the micromolar range. However, there is study in which proinflammatory activation by СTS ouabain was shown both in human macrophages (at 5–25 nM ouabain) and *in vivo* with intraperitoneal administration of ouabain to mice at a dose of 0.5 mg/kg (Chen et al., 2017). In human macrophages, ouabain has been shown to stimulate NF-κB pathway и pro-inflammatory cytokine production (TNF-α, IL6, IL-1β, monocyte chemoattractant protein-1 (MCP1)). This effect depends on a plasma membrane signaling complex that includes scavenger receptor CD36, Toll-like receptor 4 (TLR4) and Na,K-ATPase. Ouabain also triggers inflammation *in vivo*, stimulating peritonitis in mice. This process is mediated by Na,K-ATPase and CD36.

To understand the effects described, more studies are needed that compare the effect of CTSs on human and mouse monocytes/macrophages at different doses, taking into account the different sensitivity of mouse and human α1-isoforms to CTSs. Moreover, as shown above (Matsumori et al., 1997), the single action of CTS on a cell and the action of CTS in the presence of pro-inflammatory agent may be opposite. Since mouse models often used for investigation the action of CTS in the presence different pro-inflammatory factors, this may also contribute to the heterogeneity of the data.

Moreover, in the absence of the СTS-resistant α-isoform, toxic concentrations for human cells are significantly lower than for murine cells. An assessment of ouabain cytotoxicity against human macrophages (Olona et al., 2022) revealed that its concentrations in range of 50 nM < IC50 < 100 nM stimulate a dose-dependent toxic effect, but not for non-adherent human peripheral blood mononuclear cells, for which cytotoxicity does not appear up to 5 μM (Olona et al., 2022). At the same time, the density of the macrophage mannose receptor CD206, a specific marker for adipose tissue macrophages, was simultaneously decreased after mactophage treatment of ouabain. These adipose tissue-infiltrating macrophages are of particular interest due to their role in a persistent low-grade inflammation that leads to systemic insulin resistance observed in obesity (Nawaz et al., 2017). The application of ouabain (50–100 nM) to the *ex vivo* cultured white adipose tissue explants resulted in the increasing sensitivity to insulin (Olona et al., 2022). This effect can be attributed to the decreasing amount of CD206^+^ macrophages, which results in enhanced insulin sensitivity (Igarashi et al., 2018). Therefore, the therapeutic use of nanomolar concentrations of CTSs may be considered as a perspective approach for metabolic syndrome treatment, a condition marked by an infiltration and activation of macrophages in the white adipose tissue. Additionally, the ouabain treatment at concentrations 50, 100, 500 nM results in the activation of caspases 1, 3, and 7 in human macrophages, indicating cell death by apoptosis or pyroptosis. The cytotoxic effect has been found to be dependent on the cation homeostasis, as ouabain induces intracellular K^+^ depletion and accumulation of Na^+^ and Ca^2+^ (Olona et al., 2022), hinting that cytotoxicity is caused by the inhibition of Na,K-ATPase. The cell death caused by this ionic imbalance can be prevented by adding KCl to human monocyte-derived macrophages. At the same time, ouabain had a more pronounced effect on monocyte-derived macrophages compared to non-adherent peripheral blood mononuclear cell populations. Bufalin and digoxin do not exhibit such cytotoxic effects (Olona et al., 2022). The bufadienolides gamabufatalin and arenobufagin (up to 40 ng/mL, corresponding to about 100 nM) demonstrate no cytotoxicity against human peripheral blood mononuclear cells (Yuan et al., 2016). Since ouabain, bufalin and digoxin are positioned similarly in the CTS binding site of Na,K-АТPase (Kanai et al., 2021), the difference in their cytotoxicity is difficult to explain by anything but the difference in their affinity to Na,K-АТPase (Tverskoi et al., 2021). In all, the toxic effect on human mononuclear cells is caused by Na,K-ATPase inhibition and varies significantly among different CTSs owing to their distinct affinities for Na,K-ATPase.

To conclude CTSs are able to activate the pro-inflammatory response in human and mouse monocytes and macrophages, one in the presence of another activator, such as LPS, suppression of the immune response is observed. Apparently, this is the reason for the positive effect of CTSs on the survival of mice during induced inflammation, while a decrease in macrophage activity and the level of pro-inflammatory cytokines is observed. It should be noted that CTSs exhibit greater cytotoxicity towards human macrophages than towards human peripheral blood mononuclear cells. CTS effect on macrophages is contingent on the CTS type, as well as the expression levels of resistant and sensitive isoforms of Na,K-ATPase. The observed CTS effect may be primarily caused by the activation of signaling cascades, including Src kinase and NF-kB activation, whereas their inhibitory effect on Na,K-ATPase plays a secondary role.

##### 3.2.1.2 Neutrophils

CTSs have a general inhibitory effect on neutrophils that includes the inhibition of the chemotaxis and production of pro-inflammatory cytokines. It has been shown (Carvalho et al., 2019) that intraperitoneal injection of marinobufagenin (0.56 mg/kg) to mice reduces IL-1β and IL-6 production and migration of neutrophils stimulated with zymosan, the peritoneal inflammation inductor. Similar results were obtained for ouabain. The exposure of mouse neutrophils to ouabain at 1, 10 and 100 nM for 2 h reduces their chemotaxis induced by chemotactic peptide fMLP but did not prevent Akt, ERK, and JNK activation induced by zymosan (Cavalcante-Silva et al., 2021). Аt concentrations equal 1, 10 nM the effect of ouabain on chemotaxis was more pronounced and ouabain decreased p38 phosphorylation in zymosan-stimulated neutrophils. Аt a 100 nM concentration, ouabain partially inhibited Na,K-ATPase and did not alter p38-signaling. These results suggest that low doses of ouabain lead to the decrease of neutrophil migration through p38 MAPK inhibition (Cavalcante-Silva et al., 2021). Similar inflammation-reducing effects have been demonstrated for 21-benzylidene digoxin (Vieira et al., 2018) and bufalin (Zhakeer et al., 2017). Importantly, synthetic digoxin derivative compound 21-benzylidene digoxin does not inhibit Na,K-ATPase activity that eliminates dangerous toxic effects, but, despite this, it effectively suppresses the oedema progression induced by carrageenan in a dose of 0.3 mg/kg (Vieira et al., 2018). Histologic analysis revealed a decrease in the number of inflammatory cells, inducible nitric oxide synthase (iNOS) expression, and tumor necrosis factor (TNF) level in the paw pads of mice. In the study of (Jacob et al., 2013) it was demonstrated that ouabain (0.56 mg/kg ouabain) is able to reduce the migration of peritoneal exudate cells in mice with *Leishmania (L.) amazonensis* infection. This infection also increases polymorphonuclear cell population. Ouabain treatment resulted in the percentage of polymorphonuclear cells to return to the baseline. The authors suppose that decreasing amount of polymorphonuclear leukocytes observed after ouabain treatment possibly reflects a decrease of neutrophil count, as it is the predominant cell population at the beginning of the inflammatory process (Jacob et al., 2013).

All this data indicate that the impaired neutrophil chemotaxis under low concentrations of CTSs is caused by changes in signaling pathways and does not correspond with decreased Na,K-ATPase activity. As indirect support of this conclusion, we should note that redox-regulated Src kinase family signaling is necessary for macrophage wound attraction and the subsequent reverse migration of neutrophils (Tauzin et al., 2014). Beside this, Src and PI2K-dependent Akt activation is involved in control of chemoattractant GABA(B)R-mediated chemotaxis in human neutrophils (Barati et al., 2015), which appears to be important for the inflammatory response (Rane et al., 2005).

It is known that TNF and IL-8 are potent inducers for NF-kB. Experiments on human cells have shown that oleandrin blocks IL-8-induced NF-kB activation in blood-derived neutrophils, macrophages, B-cells, and T-cells. At the same time, oleandrin was unable to block TNF-induced biological responses in primary cells, i.e., macrophages and neutrophils. Oleandrin (10–1,000 ng/mL) decreases membrane fluidity in neutrophils in a dose-dependent manner, and at concentrations of oleandrin equal to 100 ng/mL it is reduced by 25% (Manna et al., 2006). Ouabain can inhibit endocytosis of interleukin 8 (IL-8), a neutrophil-activating and chemotactic cytokine. Ouabain (2.5 mM) decreases receptor-mediated endocytosis of IL-8 in human neutrophil by 96% compared to control. This results in IL-8-induced migration of ouabain-treated only by less than 10% with respect to control (Ray and Samanta, 1997). Digitoxin treatment showed a trend toward reduction in sputum free neutrophil elastase and neutrophil counts, but not a reduction in sputum IL-8. In short, at a daily dose of 0.1 mg for 28 days, digitoxin did not achieve a significant decrease in sputum inflammatory markers in patients with cystic fibrosis (CF) lung disease (Zeitlin et al., 2017). Additionally, an inhibitory influence of deslanoside was shown *in vitro* on neutrophil phagocytic function of healthy subjects. The 30 min preincubation with deslanoside (2 μg/L) decreased the phagocytosis of *Saccharomyces cerevisiae* by human peripheral blood neutrophils (Muniz-Junqueira et al., 2003).

In all, CTSs can be used as anti-inflammatory and anti-edematous drugs in therapy of various pathological conditions associated with inflammatory infiltration and activation of macrophages and neutrophils (Zhakeer et al., 2017; Vieira et al., 2018; Carvalho et al., 2019; Cavalcante-Silva et al., 2021). It should be noted that the effect of CTSs on neutrophil activity is complex and cannot be considered as strictly pro- or anti-inflammatory. Still, in view of foregoing, we incline to describe their systematic effect as mostly immunosuppressive.

##### 3.2.1.3 Eosinophils

The effect of CTSs on eosinophils has not been studied. However, a study (Zhakeer et al., 2017) of bufalin effect on asthmatic response in a mouse model, has demonstrated that this CTS (in a dose 5 and 10 mg/kg) significantly attenuated hyperresponsiveness and strongly suppressed the ovalbumin (OVA)-induced increase of total amount of inflammatory cells including macrophages, eosinophils, lymphocytes, and neutrophils in bronchoalveolar lavage fluid (BALF). Exposure to aeroallergens (1% aerosol of OVA (wt/vol) in 0.9% saline) results in antigen-specific type 2 T-helper lymphocytes (Th2 cells) response and the release of OVA-specific immunoglobulin E (IgE) in serum, and accompanied by intrapulmonary production of interleukin (IL)-4, IL-5, and IL-13 by Th2 cells. Bufalin reduces the level of these interleukins in BALF and IgE in serum (Zhakeer et al., 2017). It was found that bufalin inhibit activity of key transcription factor in the modulation of acute inflammatory response and eosinophilic inflammation in this asthmatic model - NF-κB in the lung tissues. This may underlie its anti-inflammatory effects. Notably, under hyperreactive conditions there is a pronounced eosinophilic infiltration of respiratory epithelium, thus the main effects of bufalin are assumably provided by the lowered reactivity of eosinophils.

##### 3.2.1.4 Lymphocytes

###### 3.2.1.4.1 NK cells

To the best of our knowledge, the effects of CTSs on natural killer cells (NK cells) are mediated by their influence on major histocompatibility complex (MHC) signaling. The main function of MHC is to present antigen to lymphocytes, thereby enabling the recognition and removal of altered (potentially pathogenic or infected) cells. This complex signaling pathway is one of the core processes responsible for recognition and elimination of infected or foreign cells. The effects are discussed in detail in Fu et al. (2021). In summary, bufalin directly counterbalances stimulatory and inhibitory receptors on the surface of NK cells and indirectly activates NK cells by inhibiting the shedding of MICA (MHC class I chain-related polypeptide A), a stress-induced autoantigen that functions as a ligand for the killer activation receptor and is widely recognized by NK cells (Bauer et al., 1999). Its inhibited shedding is shown to enhance the cytotoxic activity of the NK cells against the hepatocellular carcinoma cell line (Fu et al., 2021).

###### 3.2.1.4.2 T helper cells

T helper cells, or CD4^+^ T cells, are key elements of adaptive immune response, which implement their function by releasing cytokines. There is contradictory data regarding CTS impact on this cell type. On the one hand, telocinobufagin dramatically enhances a type 1 T helper cells (Th1) immune response to ovalbumin (OVA) and formalin-inactivated *Salmonella typhimurium* (FIST) in mice (Wu et al., 2015). However, on the other hand, it was shown (Da Silva et al., 2019) that intraperitoneal injection of ouabain in mice significantly decreased the number of CD4^+^ T-lymphocytes in the spleen, yet the percentage and total number of lymphocytes in mesenteric lymph nodes remained the same (Da Silva et al., 2020). Additionally, bufalin was shown to reduce the levels of cytokines IL-4, IL-5 and IL-13 produced by antigen-specific T-helper lymphocytes (Th2) in BALF (Zhakeer et al., 2017).

###### 3.2.1.4.3 Regulatory T cells

Regulatory T-cells (T-regs) are a subpopulation of T cells that suppresses the immune response and maintains homeostasis. Self-tolerance T-regs have been shown to be able to suppress T cell proliferation and cytokine production and play a crucial role in preventing autoimmunity (Kondělková et al., 2010). Tumor cells exploit the immunosuppressive capacity of CD4^+^ T-regs in tumor microenvironment to evade immune surveillance and promote tumor progression (Fridman et al., 2012). However, recent studies have demonstrated that, in some diseases, CD4^+^ T-regs do not suppress the immune response, indicating functional heterogeneity of CD4^+^ T-regs (Lin et al., 2024). Thus, the study of T-regs subtypes and targeting T-regs in tumor therapy also have great importance (for rev. see Lin et al., 2024). Characterization of the heterogeneity of T-regulator subtypes and the influence of СTSs on them is important for the balanced prevention of autoimmune diseases, on the one hand, and the development of tumors, on the other. The effects of various CTSs on the functioning of T-reg lymphocytes have been widely studied. Digoxin at a concentration of 5 mg/kg injected in mice with collagen-induced arthritis, bufatolin at a concentration of 100 μg/kg injected in mice with induced Chagrin syndrome and gamabufatolin (8 ng/mL, 19 nM) *in vitro* show similar effects, including reduction of T-helper cells expressing IL-17 (Th17) and polarization and decreased production of key pro-inflammatory cytokines IL-1β, IL-6, TNF-α and IL-21 by the inhibition of retinoic acid receptor-related orphan receptor γ thymus (RORγt) translational activity (Huh et al., 2011; Lee et al., 2015; Yuan et al., 2016; Huang et al., 2020). T-regs themselves can convert into Th17 cells in the presence of IL-6 (Xu et al., 2007). One of the main functions of T-regs is regulation of immune system activity, including restriction of excessive immune responses. Activation of T-regs results in a general immunosuppressive effect. Ouabain has been shown to reduce the number of T-regs by decreasing IL-2 production by T lymphocytes in mice (Da Silva et al., 2019). Digoxin decreased the number of Th17 cells and increased the number of T-regs in mice with collagen-induced arthritis (Lee et al., 2015). However, digoxin (1 μM), strophantin and dihydroouabain also stimulated IL17A and IL17F expression and enhanced IL17 secretion in human Th17 lymphocytes (Karaś et al., 2018). Incubation with 8 ng/mL (19 nM) of gamabufatoline, which was a nearly nontoxic concentration for normal human peripheral blood mononuclear cells, effectively reduced the percentage of CD4^+^CD25^+^Foxp3^+^ regulatory T cells in mitogen-activated peripheral blood mononuclear cells (Yuan et al., 2016).

The difference between observed effects can be explained by the choice of model organisms (mice and human) with significantly different α1-subunit Na,K-ATPase affinity to CTSs. This difference illustrates that the CTS experiments carried out on rodents cannot be extrapolated to other species of mammalian including humans.

The isoformal composition of Na,K-ATPase in T-regs remains unclear (see Table 2). Thus, the possible mechanisms of CTS effect in these cells are still insupposable. The regulation of lymphocytes by CTSs will offer new and modified treatment strategies for rheumatologic and autonomic diseases such as Chagrin syndrome (Huang et al., 2020), rheumatoid arthritis (Lee et al., 2015) via promoting T-regs and suppressing joint inflammation and bone erosion.

###### 3.2.1.4.4 T cells CD8^+^ (cytotoxic T lymphocytes)

Cytotoxic T lymphocytes are T cells that eliminate foreign and infected cells via MHC signaling system. Despite their key role in adaptive immunity, the influence of CTS on this type of cells has only been described as a part of complex anti-cancer therapy. For instance, it was shown (Li et al., 2020) that everyday administration of oleandrin alone to BALB/c mice with engrafted murine breast cancer cell line EMT6 and tumor-bearing mice intraperitoneally (0.3 mg/kg and 0.6 mg/kg) suppressed the tumor growth and increased the amount of tumor-infiltrating lymphocytes including dendritic cells and CD8^+^ T cells. In another study (Rodrigues-Mascarenhas et al., 2009), it was shown that ouabain (0.56 mg/kg with 140 mg/kg sodium succinate of hydrocortisone) injected intraperitoneally slightly increased the death of immature double positive lymphocytes (CD4^+^CD8^+^) in mice compared to the effect of hydrocortisone alone. Ouabain itself had no effect on the death of these cells.

Moreover, the simultaneous administration of the antivascular agent 5,6-dimethylxanthenone-4-acetic acid (DMXAA, also known as: ASA404, Vadimezan) and digoxin suppressed the growth of melanoma tumors in murine model (Smolarczyk et al., 2018). It was established that, in mice tumors treated with this complex therapy, the macrophages M1, CD8^+^ cytotoxic lymphocytes, NK-cells and (to a lesser extent) CD4^+^ cells increased in number. Digoxin potentiated the effect of DMXAA on increasing of CD8^+^ cell population, whereas the effect of digoxin alone is relatively weak.

In paper (Xiang et al., 2019), digoxin reversed the inability of Cisplatin to trigger calreticulin exposure and HPMA (N-(2-hydroxypropyl) methacrylamide) copolymer-amplified Cisplatin (P-Cys)-induced ATP release in melanoma mice model. These complementary mechanisms induced potent immunogenic cell death that promotes dendritic cell maturation and activates CD8^+^ T cell responses. In the presence of both digoxin (2 mg/kg) and P-Cys, the CD8^+^ cytotoxic lymphocyte count increased more significantly than in the presence of P-Cys alone therefore suggesting more pronounced anticancer effect for the combined therapy. However, no data on the individual effect of digoxin on CD8^+^ cells were provided in this study.

Ouabain treatment restored CD8^+^ T-cell numbers in mice with model of sepsis consisting of cecal ligation and puncture followed by the induction of *Salmonella enterica* *Serovar typhimurium* (S.tm.) infection (Dan et al., 2015). It was shown that ouabain can reverse immunoparalysis induced by sepsis *in vitro*, *in vivo*, and in clinical samples (Dan et al., 2015).

The effects of CTS on CD8^+^ T-cells are mainly described in oncological studies in which CTS are presumed as an additional chemotherapy. Thus, it may be incorrect to extrapolate this data to healthy organisms that are not subjected to anti-cancer therapy. It is need to denote that in most of the cases described, the CTSs *per se* did not have a significant effect on the CD8^+^ cells.

###### 3.2.1.4.5 B cells

B cells are a type of lymphocytes that is known for production of specific antibodies in response to the corresponding antigene. Ouabain has been shown to affect the B cell count. Intraperitoneal ouabain administration to mice (0.56 mg/kg) for three consequtive days led to the decrease of subpopulation of mature B cells in bone marrow, spleen and peripheral blood in 24 h after last injection (De Paiva et al., 2011). Later, it was found that the number and percentage of follicular B cells in the spleen decreased at 24 h after ouabain injection for 3 days, while, on contrary, the amount of B cells in mesenteric lymph nodes increased. These effects disappeared after 48 h and were presumed to be mediated by upregulation of chemokine receptor CXCR5 expression and downregulation of expression of cell adhesion molecule L-selectin (CD62L). Therefore, ouabain may act as a regulator of the dynamics of B lymphocyte settling in peripheral organs. Curiously, it has been shown that production of total IgM и IgG in serum of ouabain-injected mice is not altered (Da Silva et al., 2016).

In the paper (Da Silva et al., 2020), it was shown that the increased survival of melanoma-bearing mice treated with ouabain was specifically associated to the maintenance of the B cells count in splenic and mesenteric lymph nodes in melanoma-bearing animals caused by ouabain administration.

Regarding bufalin, it stimulates B cell proliferation from leukemic BALB/c mice compared with the leukemic control group at doses 0.1 and 0.2 mg/kg. Notably, a stiff dose of bufalin (0.4 mg/kg) does not lead to this effect (Hammarström et al., 1978; Shih et al., 2018).

From our point of view, the contradictory effects of different doses of CTSs described for B cells are explained by inhibitory influence of high doses of CTS on Na,K-ATPase. This influence compensates the primary effects of low CTS doses, which are provided by CTS-mediated activation of signaling cascades.

#### 3.2.2 Red blood cells (erythrocytes)

Erythrocytes are cells that supply all tissues of the body with oxygen. A distinguishing feature of these cells in mammals is the absence of a nucleus and intracellular organelles, including mitochondria. Thus, in red blood cells, CTSs are unable to induce mitochondria-dependent apoptotic pathways, as it occurs in the other cells (Chou et al., 2018). Na,K-ATPase is involved in maintaining the charge constancy of the erythrocyte membrane and influences erythrocyte deformability (Radosinska and Vrbjar, 2021). Consequently, it can be presumed that CTS are able to influence these parameters.

In human erythrocytes, the α1- and α3-isoforms of Na,K-ATPase are present (Balzan et al., 2007). The pool of Na,K-ATPase on the surface of erythrocytes is scarce: the maximum number of membrane-bound ouabain binding sites, located solely on the α-subunit of Na,K-ATPase, is 228±28 per human erythrocyte (Erdmann and Hasse, 1975). This number, as well as the ouabain affinity to the receptor, remains consistent across diverse age and sex of the blood donors. The association and the dissociation rate constants for this complex were measured at 37°C and are equal to k_+1_ = 4.6 × 10^4^ M^−1^ sec^−1^ and k_-1_ = 1.4 × 10^−4^ sec^−1^, respectively. The dissociation constant (Kd) of this complex from equilibrium binding experimentsis about 0.28 × 10^−8^ M, which corresponds to calculated from Kd = k_-1_/k_+1_ = 0.3 × 10^−8^ M (Erdmann and Hasse, 1975). According another study performed on healthy Europeans, a dissociation constant for ouabain:Na,K-ATPase complex in human erythrocytes is 1.30 ± 0.17 × 10^−8^ M (Raccah et al., 1996).

Based on these data, it can be assumed that, the rapid inhibition of Na,K-ATPase in the red blood cells, concomitant with an increase of 1.6–2 times in the level of endogenous СTSs in the blood is attributable to the low Na,K-ATPase content and the presence of highly affine α3-isoform (Emelyanov et al., 2019; Fedorova et al., 2019). In particular, the heightened circulation blood volume (hypervolemia) caused by intravenous administration of 0.9% NaCl saline solution results in enhanced secretion marinobufageninby more than 1.6 times, inhibiting Na,K-ATPase transport activity by 1.67 times (Emelyanov et al., 2019). The antibodies against marinobufagenin prevent this effect. Moreover, a similar effect has been observed in rats. A twofold increase of endogenous marinobufagenin levels in rats with inducted type 2 diabetes mellitus leads to a 30% decrease in erythrocyte Na,K-ATPase activity. Antibodies against marinobufagenin also abolish this effect (Fedorova et al., 2019). Thus, even a not very significant excess of physiological concentration (about twofold change) of blood CTSs can decrease the activity of Na,K-ATPase in red blood cells.

As demonstrated in (Zhang et al., 2018), the lifespan of red blood cells is reduced in chronic kidney disease (CKD), a disease accompanied by increased CTS levels in the blood (Orlov et al., 2021). A prevailing hypothesis asserts that CTS-induced inhibition of Na,K-ATPase, as well as CTS-induced signaling, which lead to elevating ROS levels, reduce deformability and lifespan of erythrocytes, thereby playing a pivotal role in the anemia progression in CKD (Maxwell et al., 2021). In this regard, it should be noted that analysis of clinical usage of exogenous CTS digoxin has revealed the increased risk of anemia among patients taking it (Lin et al., 2017).

Experiments with washed human erythrocytes in Ringer’s solution allowed for the reduction of CTS concentrations below physiological level, revealing the effect of Na,K-ATPase activation in the absence of endogenous CTS (Balzan et al., 2007). Additionally, the effects of digoxin, ouabain and ouabain-like factor (OLF) (10^−11^–10^−7^ M) on the Na,K-ATPase transport activity were investigated by estimating of ^86^Rb uptake. After 3 h of incubation at CTSs concentrations above 10^−9^, the activity of Na,K-ATPase decreased. Conversely, at ouabain and OLF concentrations below 10^−9^ M, Rb^+^ uptake was significantly stimulated, reaching its maximum rate of about 18% at 10^−10^ M (Balzan et al., 2007). At the same time, digoxin demonstrated no stimulatory effect on the Na,K-ATPase activity. The observed inefficacy of digoxin may be attributed to its lower affinity for Na,K-ATPase containing α1-isoform in comparison to that of ouabain (Tverskoi et al., 2021). The authors suggest that the activation effect of Na,K-ATPase may be associated with the presence of the α3-isoform in erythrocytes (Balzan et al., 2007), which is consitent with the fact that the α3-isoform shows higher affinity for CTSs than the α1-isoform (Table 1).

Throughout their lifespan, red blood cells are subjected to numerous stresses, i.e., fluctuations in oxygen level, mechanical, oxidative and metabolic stress. This may eventually alter their response to CTSs. For instance, CTS binding induces activation of Src kinase, but in erythrocytes, Src family kinases can also be activated by oxidative stress (Serafini et al., 2005). Treatment of erythrocytes with the oxidative stress inducer diamide leads to hemolysis of erythrocytes and activation of Src kinase, as well as p38 MAPK, which is also shown to be activated by Src kinase. In turn, inhibition of p38 MAPK prevents hemolysis (Hazegh et al., 2022). Thus, CTS-mediated Src kinase activation may analogously contribute to erythrocyte hemolysis. Moreover, based on these data, one can suggest that Src kinase activation under oxidative stress may interfere with CTS-mediated Src kinase activation. Thus, on the one hand, Stc-dependent signaling can be altered under conditions of oxidative stress, and, on the other hand, CTS can influence the state of erythrocytes under conditions of oxidative stress. The redox status of erythrocytes undergoes significant changes under various stress conditions including hypoxia (Fenk et al., 2022) and metabolic stress (Zaripov et al., 2023). Under hypoxic conditions, activating phosphorylation of Src kinase is increased, and Src kinase does not responds to CTS (Petrushanko et al., 2017). We believe that this is due to the fact that glutathionylation of Na,K-ATPase, which increases during hypoxia, may disrupt its interaction with Src kinase, which has been shown by molecular modeling (Petrushanko et al., 2017). In all, one can conclude that the glutathionylation of Na,K-ATPase caused by altered redox status under hypoxia (Petrushanko et al., 2012) or oxidative stress is able to disrupt Src kinase and Na,K-ATPase interaction, leading to impaired activation of Src kinase by CTSs. Consequently, various physiological stresses, which erythrocytes are exposed to (e.g., metabolic stress, temperature stress, mechanical stress, and deoxygenation), may affect their response to circulating CTS in blood by affecting RBC redox status.

Metabolic stress associated with glucose deficiency merits particular consideration. For instance, glucose deficiency has been shown to induce a decrease in the glutathione levels (Nemkov et al., 2020; Zaripov et al., 2023) and stimulate glutathionylation of haemoglobin (Hb) (Zaripov et al., 2023). Furthermore, it has been shown that both types of diabetes mellitus (type 1 and type 2) (Silbert et al., 2018; Szadkowska et al., 2018), damage of hypophysis (Vega-Cano et al., 2021), sepsis (Wang et al., 2021) surgical intervention (Salehi et al., 2018) chronic hypoglycemia may occur. In diabetic patients, the development of neuropathy is strongly related to decreased Na,K-ATPase activity, caused by accumulation of sorbitol and fructose simultaneously with the depletion of myonositol (Flier et al., 1987). In neurons of patients with diabetes, Na,K-ATPase activity is reduced (Das et al., 1976). Due to the great importance of the sodium-potassium gradient for neuronal function, improving Na,K-ATPase function plays an important role in the treatment of neuropathy (Dhanapalaratnam et al., 2025). Since a correlation between Na,K-ATPase activity in RBC membranes and the conduction velocity innerve fibers was observed (Raccah et al., 1996), the following research with erythrocytes was conducted. In erythrocytes of Caucasian patients with insulin-dependent diabetes and healthy North African subjects, which are predisposed to diabetic neuropathy, Na,K-ATPase activity is reduced by 30%, compared to healthy Caucasian subjects (Raccah et al., 1996). Additionally, the number of ouabain binding sites on erythrocytes, which indicates the amount of Na,K-ATPase on the red blood cell surface, was reduced in patients with insulin-dependent diabetes and healthy North African subjects compared to healthy Caucasian subjects. However, for healthy North African subjects, as well as for healthy Caucasian subjects, the Na,K-ATPase activity correlates with the maximum number of ouabain binding sites on erythrocytes, whereas no such correlation has been found for patients with insulin-dependent diabetes. The dissociation constants for Na,K-ATPase:ouabain complexes did not differ significantly in all three groups. Thus, the authors suggest that the constitutional decrease in Na,K-ATPase activity in healthy North African subjects corresponds to a quantitative defect, but the absence of the correlation between the decrease in Na,K-ATPase activity and the number of ouabain binding sites in diabetic patients corresponds to a qualitative defect. This is possibly caused by the decreased availability of ouabain binding sites on erythrocytes due to the alterations in structure of the lipid membrane (Raccah et al., 1996). Thus, it can be expected that RBC of patients with diabetes will respond differently to CTSs than RBC of healthy volunteers.

All in all, despite the scarcity of Na,K-ATPase in erythrocytes, it is crucial for their functionality and viability, particularly due to its role as a receptor for CTSs. This must be taken into account while prescribing CTSs as drugs, as well as while investigating the influence of pathological alterations of endogenous CTSs levels on the body.

#### 3.2.3 Clotting and platelet (thrombocyte) dependent effects

Thrombocytes (platelets) are a part of the system that causes blood clotting, or coagulation, in response to bleeding. Platelets are derived from the large nuclear cells - megakaryocytes, which are found in the bone marrow. Platelets are only 2–3 microns in size, do not contain a nucleus and play an important role in the hemostasis system, and secondary role in innate immunity and angiogenesis [for rev. see (Thon and Italiano, 2012)]. Numerous studies have shown that some CTSs have a procoagulant effect (Chirinos et al., 2005). This is probably due to the direct action of CTSs on platelets. Patients with atrial fibrillation taking digoxin have increased levels of platelet activation compare to patients compared with patients not taking (Chirinos et al., 2005). Treatment of patients with digoxin leads to increase of the expression level of P-selectin (CD62P, a marker of platelet activation) in human platelets and platelet-leukocyte conjugates (Chirinos et al., 2005).

Direct correlation was found between serum digoxin level in the patients and marker reflecting platelet activation. Moreover, digoxin (2.4 ng/mL, 3 nМ) induced calcium mobilization, PAC-1 (procaspase-activating compound 1) expression and platelet aggregation in patients with atrial fibrillation but not in healthy subjects. Lower digoxin concentrations of 0.6–1.2 ng/mL (0.76–1.5 nM), which are closer to physiological, did not have this effect. To mention that patients with AF have higher basal platelet activation than healthy patients (Pastori et al., 2018). After pretreatment of thrombocytes from healthy subjects with collagen to mimic the preactivation *in vitro*, digoxin induced calcium mobilization, arachidonic acid release, TxB2 biosynthesis, platelet PAC-1 and P-selectin expression, and promoted platelet aggregation in a dose-dependent manner. Antibodies to digoxin abolished these effects. The phosphorylation of calcium-bound phospholipase A2 is involved in this process of the platelet aggregation (Pastori et al., 2018). However, procoagulant activity was not observed in healthy volunteers taking digoxin (at a dose of 0.6 mg on day 1, 0.4 mg on day 2, then 0.1 mg daily for 10 days), and no difference between digoxin and placebo was observed (Pettersen et al., 2002). Notably, ouabain also has procoagulant activity. It has been shown that treatment of human platelets *in vitro* with ouabain (20–200 μM for 20–60 min) is associated with dose-dependent intracellular accumulation of sodium, generation of a weak calcium signal, and induced procoagulation (Tomasiak et al., 2007). Inhibition of platelet Na,K-ATPase by ouabain leads to an increase in intracellular Na^+^. The increase in Na^+^ and associated cell swelling may in turn lead to phosphatidylserine exposure and increased membrane curvature, resulting in procoagulant activity. According to thromboelastography data, increasing ouabain concentration to nanomolar range (50–5,000 nM, 15 min) in whole human blood results in an increasing rate of clot formation initiated by contact and high extracellular calcium concentration. (Tomasiak et al., 2007). The procoagulant effect induced by ouabain is dose- and time-dependent, less pronounced than the collagen-induced response, and significantly reduced in the absence of extracellular Na^+^ or in hyperosmolality (Tomasiak et al., 2007). Thus, the procoagulant effect of СTSs is associated with the inhibition of Na,K-ATPase pump activity.

All the effects of different CTSs on blood cells are summarized in Supplementary Table 1.

## 4 Peculiarities of CTS effects revealed in blood cells and future directions

Different effects of various CTSs on the same cell type (Supplementary Table 1) may be foremost associated to the different selectivity to Na,K-ATPase α-isoforms, different binding and inhibition constants for different CTSs (Klimanova et al., 2015; Tverskoi et al., 2021), as well as to the fact that their binding to Na,K-ATPase results in stabilization of different conformations of the enzyme (Katz et al., 2010; Klimanova et al., 2015). The analysis performed in this review demonstrate that all blood cells contain not only housekeeping α1-isoform, but also tissue-specific isoforms α3- and, to a significantly lesser extent, α2-isoforms (Table 2), providing the huge variety of possible CTS effects combined.

We would like to point out the difference between CTS effects on rodents and humans. First of all, we would like to remind that CTS affinity to mice α1-subunit of Na,K-ATPase is 1,000 times lower than to human one, yet the affinities of α2- and α3-subunits are the relatively close in magnitude. This CTS-resistant α1-isoform of Na,K-ATPase in rodent cells does not have any known analogues in human cells. Furthermore, at the doses of CTSs used in experiments on mice (about 0.56 mg/kg), the maximum concentration in the blood lies in the micromolar and submicromolar range, which is acceptable for rodents due to the presence of CTS-resistant α1-subunit yet lethal to other mammals, including humans, for which only nanomolar concentrations are acceptable. Therapeutic dose of digoxin for human is 0.25 mg (0.0037 mg/kg at mean weight 67 kg) (Heidenreich et al., 2022). This means that the effects of CTSs in humans and mice are studied in different concentration ranges. Therefore, it is difficult to extrapolate effects of СTSs obtained in rodents to humans. In all, mouse and rat models have significant limitations for the studies regarding CTSs, their effect on humans and their role in the pathogenesis of human diseases.

In order to compare the CTSs effects on blood cells, their lifespan in blood, which is measured as elimination half-life (T_1/2_), should be taken into consideration. The study of the pharmacokinetics of digoxin in healthy volunteers showed that, after oral administration or intravenous injection of 0.6 and 1.2 mg of digoxin (from 0.0089 mg/kg to 0.0179 mg/kg, mean weight of patients 67 kg), maximal mean concentrations of these CTSs in plasma were 4.3 and 9.5 ng/mL (5.5 nM and 12 nM correspondingly) observed in 40 min (Hinderling and Hartmann, 1991). Intravenous injection of digoxin to mice in dose 0.1 mg/kg stimulated the increase of its concentration up to 350 ng/mL (448.7 nМ) in serum in 30 min (T_1/2_ = 90 min) (Kawaguchi et al., 2004). Intravenous injection of bufatalin to mice in dose 6 mg/kg increased its levels in plasma up to 5 μg/mL (11 µМ) in 5 min, but it bounced back quickly due to lower T_1/2_ = 28.6 min) (Yu and Hou, 2011). After peroral administration of resibufagenin (1.31 mg/kg), bufalin (1.72 mg/kg), gamabufatalin (0.35 mg/kg), arenobufagin (0.57 mg/kg) to mice, the maximum concentration in plasma were 654.33 ng/mL, 1786.24 ng/mL, 155.62 ng/mL, 471.83 ng/mL, respectively. The values of T_1/2_ ranged from 0.99 h to 2.47 h (Huang et al., 2015). Doubtlessly, the difference in elimination half-life of various CTSs may be responsible for the difference between their effects on blood cells.

Blood cells are exposed to varying levels of oxygen as they circulate. In human arterial blood, O_2_ partial pressure is equal 12,7 kPa (11.1 for rat blood), however, in venous blood this value is significantly lower than 6.3 kPa (5.3 for rat blood), in some tissues it is much lower (2.53 kPa for rat cortex brain) (Bogdanova et al., 2006) and may be even lower at some pathological conditions. Since main effects of CTSs in blood cells are caused by the activation of signal cascades, as a result of receptor function of Na,K-ATPase, which depends on the level of cell oxygenation (Lakunina et al., 2017; Petrushanko et al., 2017), it is suggestable that CTS effect on the cells differs in main bloodstream and in peripheral blood. This alteration may be especially important for immune cells that migrate from blood stream to tissues. Additionally, hypoxia may affect the cellular effect of CTSs, and, in turn, CTSs, particularly digoxin, may inhibit the synthesis of HIF1-α (Zhang et al., 2008; Lin et al., 2009). The possible role of CTSs in the adaptation of tissues to hypoxic conditions is currently debatable (Baloglu, 2023). In particular, it remains unclear whether ouabain or other CTS mediate hypoxic stress-dependent regulation of Na,K-ATPase by inhibiting HIF in ischemic heart disease (Baloglu, 2023). Besides, it should be taken into account that, in cardiovascular disease, the heart is more sensitive to the effects of ouabain due to a decreasing count of membrane Na,K-ATPase and that the use of exogenous CTS in heart failure in presence of elevated plasma levels of endogenous ones may further exacerbate disease progression through various signaling cascades (Baloglu, 2023). Thus, the estimation of the impact of different oxygen levels on altering the CTS effects on blood cells requires further investigations.

All the aforesaid examples of CTS effects on blood cells show that CTS effects of practical interest are not associated to Na,K-ATPase inhibition (excluding their effect on platelets) but are caused by the activation of Na,K-ATPase or signal cascades. Since the effects of CTSs on leukocytes and red blood cells are mediated by the activation of signal cascades, it is important to design artificial CTSs that can activate signal cascades without toxic Na,K-ATPase inhibition effect.

## 5 Discussion

**FIGURE 3:**
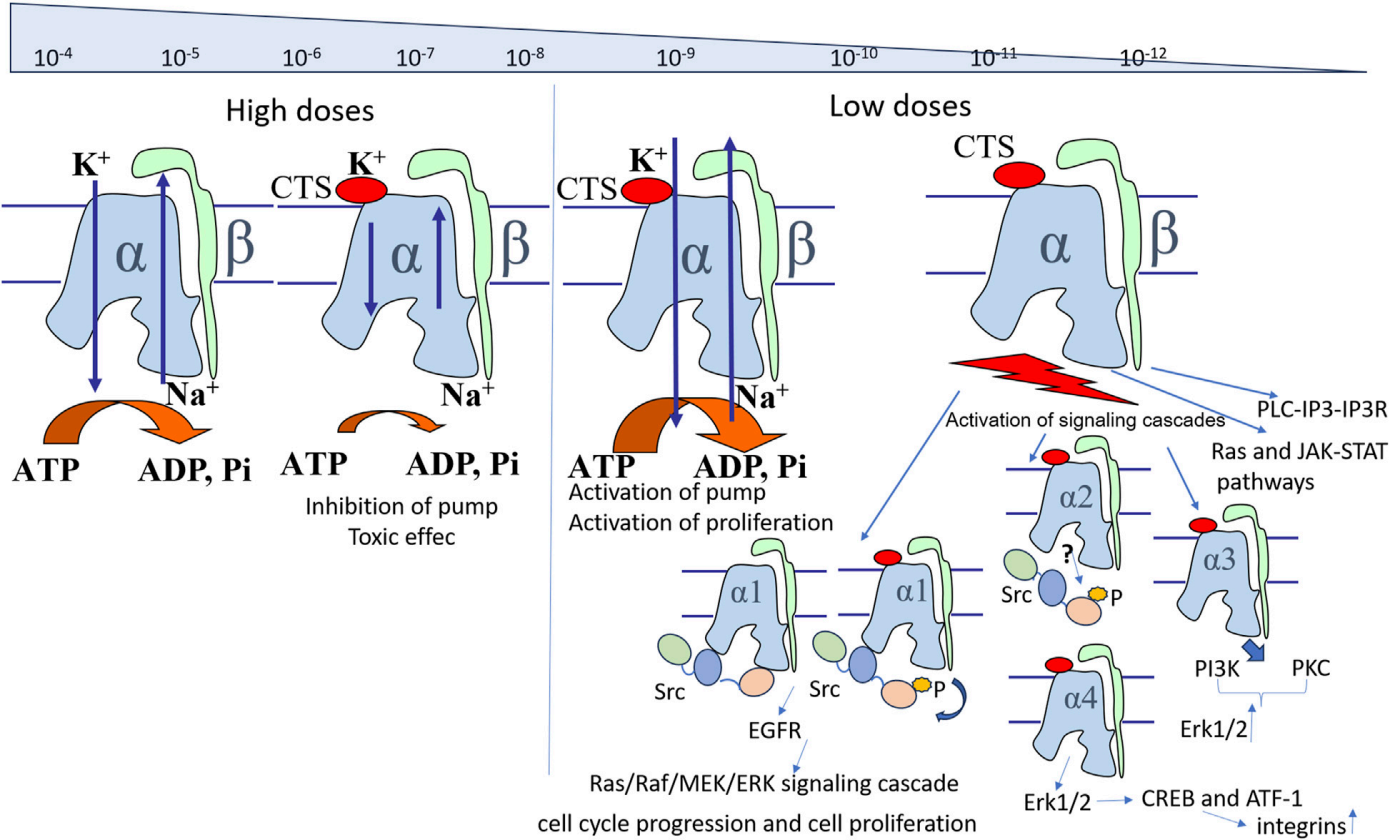
Scheme of the CTS effects on the Na,K-ATPase functioning (This scheme created manually in the MS PowerPoint). A description of the scheme is given in the text.

This review summarizes (Supplementary Table 1) and considers the effect of CTSs on blood cells in the light of the known mechanisms of their action on mammalian cells (Figure 3). Cardiotonic steroids entering the bloodstream can be exogenous (ingestion of drugs or poisonous plants) or endogenous (produced by the body itself). Initially, the influence of exogenous CTSs was considered mainly in the scope of their therapeutic target, i.e., the heart, and the analogous impact of endogenous CTSs on vascular tone. Now, we propose to direct the focus towards the effect of these compounds on blood cells, which are among the first to encounter altered CTS levels. The data presented in this review indicates that the blood cell response to alterations in CTS levels may mediate numerous systematic changes and therefore should not be ignored.

Cardiotonic steroids are important regulators of cellular processes, with complex mechanisms of action. In recent years, their role in distinct pathologies has been actively studied in various cell lines. It is known that the human body maintains a rather low but constant rate of CTS synthesis (plasma levels in population studies were 111–200 pmol/L) (Wang et al., 2003; Manunta et al., 2009) stated that extraadrenal or dietary sources are not sufficient to maintain stable plasma concentrations of endogenous ouabain. Additionally, the adrenal glands are enriched by endogenous ouabain in many mammals, and its content in tissues remains remarkably constant under different conditions in concentrations insufficient to inhibit Na,K-ATPase (Manunta et al., 2009; Lakunina et al., 2017). Plasma CTS levels in healthy volunteers are found in the subnanomolar range (Wang et al., 2003; Orlov et al., 2021). However, in a number of diseases and pathologies, their concentration increases severalfold, suggesting their adaptogenic and regulatory role that enable the organism to withstand acute pathological conditions. This assertion is supported by the fact that CTS levels are increased in diseases that affect numerous organs and systems, including those connected with the circulatory system and water-salt metabolism. These are, for instance, arterial hypertension, myocardial infarction, heart failure, pre-eclampsia, chronic renal failure and aldosteronism (Orlov et al., 2021). A common feature among these diseases is the impaired tissue perfusion and the consequently increasing burden on the cardiovascular system.

The pathways of influence of different CTS concentrations on the state of cells upon binding to Na,K-ATPase are presented in Figure 3.

The effect of CTSs depends on their concentration (Figure 3). At high concentrations comparable to the enzyme inhibition constant or higher (High Doses), interaction of CTSs with Na,K-ATPase inhibits the transport activity of the enzyme, adversely affecting cell viability, and leads to the development of toxic effects at prolonged exposure. At lower concentrations (Low Doses) (5 × 10^−7^ − 5 × 10^−10^ M) (Orlov et al., 2021), activation of signaling cascades is observed (Paula et al., 2005; Pratt et al., 2018; Lopina et al., 2020). Activation of signaling cascades is mostly caused by the Src kinase interaction with Na,K-ATPase (Li and Xie, 2009; Banerjee et al., 2015). Src kinase has been shown to interact directly to α1-subunit, and this interaction is disrupted by CTS binding to Na,K-ATPase that leads to autophosphorylation of Src and its activation (Li and Xie, 2009; Karpova et al., 2010; Petrushanko et al., 2017; 2022). In turn, Src kinase induces EGFR activation (Chen et al., 2018), resulting in Ras/Raf/MEK/ERK signaling cascade and affecting the regulation of cell cycle and proliferation (Škubník et al., 2021). CTS binding to α2-subunit also leads to Src kinase activation, while α3 induces Erk1/2 activation in a Src-independent way mediated by PI3K and PKC (Karpova et al., 2010; Madan et al., 2017). α4-subunit may also activate Erk1/2 signaling cascades and stimulate integrins expression through CREB and ATF-1 (Upmanyu et al., 2016). Ras and JAK-Stat molecules (Farooqi et al., 2023), as well as PLC-IP3-IP3R (Xie and Cai, 2003; Reinhard et al., 2013) pathways, can also be activated via Na,K-ATPase. Lower concentrations of CTS, reaching about 3 × 10^−10^-10^−11^ М (Orlov et al., 2021), stimulate Na,K-ATPase transport activity (Oselkin et al., 2010; Tverskoi et al., 2016; Lopina et al., 2020).

The analysis of the available data suggests that erythrocytes, the most prevalent blood cells,– exhibit a responsiveness to even minor fluctuations of endogenous CTSs (Emelyanov et al., 2019; Fedorova et al., 2019), which is probably due to the low amount of Na,K-ATPase and the presence of the high-affinity α3-isoform (Erdmann and Hasse, 1975; Balzan et al., 2007). It is currently considered that a significant and prolonged change in blood CTS levels due to chronic kidney disease (Zhang et al., 2018; Maxwell et al., 2021; Orlov et al., 2021) or digoxin administration (Lin et al., 2017) may be responsible for the decreased lifespan of red blood cells. In the context of CTS impact on platelets, a direct dose-dependent platelet activation, associated with the inhibition of platelet Na,K-ATPase, has been revealed. This effect has been shown both *in vitro* (for ouabain) (Tomasiak et al., 2007) and *in vivo* (for digoxin), particularly in patients with cardiovascular pathology (Pastori et al., 2018). Further, in relation to the leukocytes, the impact of CTSs on these cells has been most extensively studied (Supplementary Table 1). CTSs exert a regulatory effect on the cells of the immune system, preferentially preventing the development of hyperergic (superphysiological) responses and contributing to the limitation of inflammatory damage to organs and tissues. Importantly, the effects observed in murine models containing the СTS-resistant α1-isoform and in human cells expressing the СTS-sensitive α1-isoform differ substantially. For example, in response to the CTSs, the secretion of proinflammatory cytokines in monocytes and macrophages is heightened in human models (Raviprakash and Manna, 2014; Teixeira and Rumjanek, 2014) and inhibited in murine ones (Carvalho et al., 2019; Gao et al., 2022), which is primarily associated with the activation of signal cascades (Khalaf et al., 2019; Gao et al., 2022). As demonstrated by the presented data (Supplementary Table 1), СTSs are able to influence almost all types of leukocytes, changing their production of cytokines. Depending on the cell type, concentration, and CTS, both inhibitory and activating effects were observed (Supplementary Table 1). In some cases, it has been described that the activating effect of CTS alone is replaced by an inhibitory effect if the cells are additionally activated by another factor.

The presumable reasons for the different effects of CTSs on distinct cell types are the expression of tissue-specific isoforms of Na,K-ATPase and the peculiarities of the regulation of signaling cascades in different cell types. When considering the effect of CTSs on various blood cells (Supplementary Table 1), it is primarily important to draw attention to the isoform composition of the α-subunits in the cells (Table 2), since isozymes containing different α-isoforms differ in their affinity for CTSs and ions (Table 1) and ability to induce different signaling cascades (Figure 3). If present, the α1-resistant isoform, which is characteristic of rodents, should be a matter of a particular importance, since it radically changes the resistance of cells to CTSs and, consequently, the СTS-dependent response itself. It is also important to consider the type of CTSs, given that the affinity of different CTSs to the identical isozymes of Na,K-ATPase may vary significantly. The dose-dependent response of Na,K-ATPase to different concentrations of СTSs is schematically shown in Figure 3. It is suggested that the probable reasons for the increase in Na,K-ATPase activity at low concentrations, which precedes its inhibition, may be associated with the displacement of an endogenous inhibitor from a tetrameric form of the Na,K-ATPase (Blaustein and Hamlyn, 2024). Authors suggested that this endogenous inhibitor is an endogenous CTSs and suppose that in this case, it is clear why the activation data varies so much. After all, the level of endogenous CTSs depends on the state of the body, various effects, such as hypoxia, can alter it’s levels and, depending on sample preparation, eCTSs can dissociate, or somehow change its properties. Moreover, the effect of different CTSs on Na,K-ATPase can be different; in particular, authors discussed antagonism digoxin and ouabain (Blaustein and Hamlyn, 2024). Indeed, binding of ouabain and marinobufagenin leads to different structural changes in Na,K-ATPase (Klimanova et al., 2015), and ouabain can displace marinobufagenin from the complex with Na,K-ATPase (Klimanova et al., 2015) due to the higher binding constant with ouabain in the E2P conformation. Since marinobufagenin is present in blood plasma (Orlov et al., 2021) and, unlike ouabain, readily binds to Na,K-ATPase in any conformation (Klimanova et al., 2015), we assume that marinobufagenin gets isolated with the protein. Further, when exogenous ouabain is injected, marinobufagenin gets displaced, which possibly mediates the effects described in the review.

The experimental data presented in the review indicates that, when considering the effect of CTSs on blood cells, it is necessary to assess the overall state of the body, particularly the parameters such as blood oxygenation, blood glucose level, and physical activity. In particular, as aforementioned, all blood cells, moving in the bloodstream, are subjected to the alterations in oxygen levels, with venous blood exhibiting lower oxygenation than arterial blood. The factors such as decreased oxygen in the surrounding air (hypoxia) or pulmonary pathologies further reduce the level of venous blood oxygenation, which alters the cellular response to CTSs. Due to the sensitivity of the Na,K-ATPase:Src kinase cascade to the redox state, the oxygen deficiency drastically alters the effect of CTSs on cells, which should be taken into account when considering the mechanism of their action under hypoxic and ischemic conditions (Lakunina et al., 2017; Petrushanko et al., 2017). Concurrently, deoxygenation results in the increasing CTS levels in blood (Bagrov et al., 1998; De Angelis and Haupert Jr, 1998; Orlov et al., 2021). A presumable protective role of CTSs in hypoxia (Bagrov et al., 1998; Oselkin et al., 2010; Lakunina et al., 2017) is still a subject of debates (Baloglu, 2023).

It is also necessary to take into account the level of glucose in the blood, a factor that is especially important for erythrocytes, for which glycolysis is the main source of ATP. Under metabolic stress associated with a glucose deprivation in red blood cells, a change in their redox status is observed (Nemkov et al., 2020; Zaripov et al., 2023), which, as noted earlier, influences the Src-dependent signaling cascade. Physical exercises also affect the redox status of blood cells (Stagos et al., 2015; Xiong et al., 2018), therefore altering the Na,K-ATPase:Src kinase signaling response. Further, numerous diseases that alter the redox status of blood cells, such as chronic kidney disease (Garavaglia et al., 2022), diabetes (Sánchez-Gómez et al., 2013), and Alzheimer’s disease (Martínez De Toda et al., 2019), obviously influence the blood cell responsiveness to CTS. Moreover, it is important to note that the basal level of endogenous CTSs able to influence on the effect of exogenous CTSs (for rev see (Blaustein et al., 2022)). In particular, ouabain-digoxin antagonism has been described (Blaustein et al., 2022). For example, digoxin reverses ouabain-induced hypertension, effects of ouabain on cardiac responses induced by digoxin, contractions of digoxin-treated muscul, etc (Blaustein et al., 2022). Thus this complex regulation is a subject that merits future research.

Additionally, it is important to take into account another component that is constantly present in the blood, which probably affects the CTS effect. This is beta-amyloid peptide. Blood cells are shown to produce and bind beta-amyloid, which levels in the bloodstream typically approach those of CTS (Kiko et al., 2012; Lan et al., 2015; Sun et al., 2020; Orlov et al., 2021). The main source of blood-produced beta-amyloid are platelets. Leukocytes also express beta-amyloid precursor protein (APP), but Aβ itself appears to be produced by this cell type in rather small quantities. Erythrocytes do not have APP, but are capable of binding beta-amyloid, which affects their properties (for rev see (Wojsiat et al., 2017)). In Alzheimer’s disease (AD), the percent of beta-amyloid-bound red blood cells is increased (Lan et al., 2015). Beta-amyloid, a peptide that plays an important role in the pathogenesis of Alzheimer’s disease, binds to the extracellular part of Na,K-ATPase likewise CTSs. At high concentrations, it inhibits Na,K-ATPase (Dickey et al., 2005; Kreutz et al., 2013; Petrushanko et al., 2016), whereas its low concentrations lead to Na,K-ATPase-mediated activation of Src kinase (Petrushanko et al., 2022). Although the exact binding sites of beta-amyloid and CTSs are different (Adzhubei et al., 2022), the similarity of their effects on Src-kinase activation may imply the interdependence of beta-amyloid and CTS effects on cells.

Importantly, the fluctuations in endogenous CTS levels observed in various neurodegenerative diseases, including AD and Parkinson’s disease, have been demonstrated in numerous studies (Orellana et al., 2016; Lopachev et al., 2018; Fedorova et al., 2020). One of the most significant effects of CTSs is their capacity to mitigate neuroinflammation, a hallmark of these diseases. In murine models of AD, the level of marinobufagenin in serum is decreased, whereas its exogenous administration has been shown to mitigate AD-associated neuroinflammation by reducing IL6 level in brain (Fedorova et al., 2020). In a different murine model of AD (APP/PS1), a sex-specific hypothalamic neuropathology has been shown (Qi et al., 2024), which is especially intriguing given that hypothalamus is assumed to be responsible for CTS synthesis in the brain. Weidemann et al. showed the production of endogenous CTS in murine hypothalamus (Weidemann et al., 2004). Alterations of brain-derived CTS synthesis in AD weres not shown yet, nevertheless, the production of sex hormones typically synthesized by hypothalamus is decreased even in early stages of AD, which is possibly associated with its volume reduction (Laurell et al., 2024). At the same time, it is known that the interaction of endogenous CTSs with the α2- and α3-isoforms of Na,K-ATPase has been demonstrated to modulate learning and memory, as well as mood and behavior (Blaustein and Hamlyn, 2020). Thus, the administration of exogenous CTSs may diminish the symptoms of AD, restoring their neurohormonal activity and attenuating neuroinflammation.

In all, fluctuations of endogenous CTSs in pathological conditions, as well as the administration of exogenous CTSs, potentially alter the functionality of erythrocytes and leukocytes and induce the activation of platelets. The influence of CTSs on blood cells enables them to perform the role of sophisticated adaptive regulators, thereby providing the formation of appropriate systematic responses to both external and internal stimuli. Acute changes in the level of СTSs can enable the organism to withstand short-term adverse conditions; nevertheless, a prolonged high-dose exposure can exert numerous toxic effects.

## 6 Conclusion

CTSs are complex physiological regulators that provide the maintenance of homeostasis of the organism under various pathological conditions. CTSs exert specific effects on different blood cells. The expression levels of different Na,K-ATPase isoforms with various properties and sensitivity to CTSs, determine the general CTS effect on each cell type of blood. At high concentrations, comparable or higher than the dissociation constant of the ubiquitous α1-isoform of Na,K-ATPase, CTSs exert an inhibitory effect on ion transporter, resulting in a cytotoxic effect at long-term exposure. At low concentrations (nanomolar and subnanomolar), insufficient to inhibit the pump function, the signaling function of CTSs and/or Na,K-ATPase overactivation are observed, stimulating cell proliferation and altering cell functionality. Consideration of endogenous CTSs as markers of compensation in various pathological conditions will allow creating approaches for promising diagnostic technique.
