# Supplementary material for: Mechanisms mediating effects of cardiotonic steroids in mammalian blood cells
Source: Front Pharmacol. 2025 Mar 24;16:1520927. doi: 10.3389/fphar.2025.1520927 (PMC11973394; doi:10.3389/fphar.2025.1520927)
Supplement: Supplementary file 1 [file Table1.docx]

Supplementary Material

# Supplementary Tables

Supplementary Table 1. Cellular and systemic effects of cardiotonic steroids on different blood cells

| **Cell type** | **CTS, C/B(+/-)** | **Cellular and systematic effects** |
| --- | --- | --- |
| Erythrocytes | OBN, C(+) | (1) Play an important role in the development of anemia in chronic kidney disease (Maxwell et al., 2021) through the increase in ROS levels, impaired erythrocyte deformability and lifespan reduction |
|  | MBG, B(-) | (1) Released in response of hypervolemia (Fedorova et al., 2019)  (2) An increase in endogenous marinobufagenin in rats with inducted 2 diabetes mellitus leads to a 30% decrease in erythrocyte Na,K-ATPase activity (Fedorova et al., 2019) |
|  | DGX, С(+) | (1) Did not stimulate the Na,K-ATPase activity (Balzan et al., 2007) |
| Neutrophils | OBN, С(+) | (1) Inhibiting migration (Ray and Samanta, 1997; Cavalcante‑Silva et al., 2021)  (2) Reducing chemotaxis induced by chemotactic peptide fMLP (Cavalcante‑Silva et al., 2021)  (3) Reducing the migration of peritoneal exudate cells in mice with Leishmania (L.) amazonensis infection (Jacob et al., 2013)  (4) decreasing amount of polymorphonuclear leukocytes observed after ouabain treatment at the beginning of the inflammatory process (Jacob et al., 2013)  (5) Inhibit endocytosis of interleukin 8 (IL-8) (Ray and Samanta, 1997) |
|  | MBG, B(-) | (1) Inhibiting migration (Carvalho et al., 2019) |
|  | 21‑Benzylidene digoxin, C(+) | (1) Inhibiting of TNF–α production release (anti-inflammatory and edema inhibiting effects) (Vieira et al., 2018). Decrease in the inducible nitric oxide synthase (iNOS) expression in the paw pads of mice |
|  | BUF, B(-) | (1) Attenuation of hyperresponsiveness. Decreased total number of inflammatory cells (Zhakeer et al., 2017) |
|  | Oleandrin(-) | 1. Blocks IL-8-induced NF-kB activation in blood-derived neutrophils (Manna et al., 2006) 2. Decreasing membrane fluidity in neutrophils in a dose-dependent manner (Manna et al., 2006) |
| Eosinophils | BUF, B(-) | (1) Attenuation of hyperresponsiveness. Decreased total number of inflammatory cells (Zhakeer et al., 2017) |
| Macrophages | OBN, C(+) | (1) Reduced TNF-α and IFN-γ levels (Jacob et al., 2013)  (2) Cell death of monocyte-derived macrophages [100] (dose-dependent toxic effect on human macrophages)  (3) Produced higher levels of IL-1 β and TNF- α, IL-10 and VEGF. Increased expression of surface activation markers [99]  (4) Decrease of macrophage mannose receptor CD206 (marker for adipose tissue macrophages) (Nawaz et al., 2017) |
|  | MBG, B(-) | (1) Attenuation of proinflammatory cytokines (Carvalho et al., 2019) |
|  | Oleandrin, C(+) | (1) Enhanced biological responses to IL-8, without cytoxicity [102] |
|  | Resibufogenin, B(-) | Resibufogenin in LPS-stimulated macrophages reduces the production of pro-inflammatory mediators (iNOS, IL-6, MCP-1) by suppressing their transcription.(Gao et al., 2022). |
|  | Telocinobufagin, B(-) | (1) Induce oxidative burst and enhanced NF-KB activation. (Khalaf et al., 2019) |
| Monocytes | MBG, B(-) | (1) Reduced levels of proinflammatory cytokines IL-1β, IL-6, and TNF-α (Carvalho et al., 2019). |
| NK-cells | Oleandrin, C(+) | (1) Balancing stimulating and inhibitory receptors on the surface of NK cells and indirectly activates NK cells by inhibiting MICA shedding (Fu et al., 2021) |
|  | BUF (B-) | Counterbalances stimulatory and inhibitory receptors on the surface of NK cells and indirectly activates NK cells by inhibiting the shedding of MICA (MHC class I chain-related polypeptide A) (Bauer et al., 1999) |
| T-killers | OBN, C(+) | (1) Did not alter the percentage and absolute numbers of CD8+T lymphocytes (Da Silva et al., 2020a) |
| T-helpers | OBN, C(+) | (1) Reduced number of CD4+ T-lymphocytes in the spleen (Da Silva et al., 2019)  (2) Did not alter the percentage and absolute numbers of CD4+T lymphocytes (Da Silva et al., 2020a) |
| T-helpers  T-regs | Telocinobufagin, B(-) | (1) Enhancing a Th1 immune response to control intracellular infections (Wu et al., 2015) |
|  | OBN, C(+) | (1) Reduced number by decreased Il-2 production by T-lymphocytes (Da Silva et al., 2019)  (2) Did not alter the percentage and absolute numbers of CD4+T lymphocytes (Da Silva et al., 2020a) |
| T-regs  CD8+ cells | DihydroOBN, C(+) | (1) Upregulation of IL17A and IL17F expression and enhanced IL17 secretion (Karaś et al., 2018) |
|  | DGX, C(+) | (1) Reduced expression of proinflammatory cytokines. Can regulate Th17 and reciprocally promote Treg cells and suppress joint inflammation and bone erosion in CIA. (Lee et al., 2015)  (2) Reduced in vitro differentiation and LPS-stimulated IgG production. Suppression of joint inflammation and bone erosion in CIA (Lee et al., 2015)  (3) Upregulation of IL17A and IL17F expression and enhanced IL17 secretion (Karaś et al., 2018)  (4) Inhibiting RORγt translational activity. (Huh et al., 2011) |
|  | BUF, B(-) | (1) Inhibiting polarization (Huang et al., 2020)  (2) Inhibiting secretion of cytokines IL-17 and IFN-γ (Huang et al., 2020) |
|  | Strophantin, C(+) | (1) Upregulation of IL17A and IL17F expression and enhanced IL17 secretion (Karaś et al., 2018) |
|  | Gamabufotalin, B(-) | (1) Downregulation of the percentages of CD4+CD25+Foxp3+ Treg cells in mitogen-activated PBMCs (Yuan et al., 2016) |
|  | OBN, C(+) | (1) Induce the death of immature double positive lymphocytes (CD4+CD8+) (Rodrigues-Mascarenhas et al., 2006). |
| CD8+ cells  B cells | DGX, C(+) | (1) Inhibition of the growth of melanoma tumors in murine model (Smolarczyk et al., 2018)  (2) Reversed the inability of Cisplatin to trigger calreticulin exposure, and HPMA copolymer-amplified Cisplatin-induced ATP release in melanoma mice model (Xiang et al., 2019) |
|  | Oleandrin, C(+) | (1) Inhibite tumor growth and increase tumor-infiltrating lymphocytes including dendritic cells and T cells (Li et al., 2020) |
|  | OBN, C(+) | (1) Decrease the level of B-cells in bone marrow, spleen and peripheral blood in 24 hours Immunobiology. (de Paiva et al., 2011)  (2) Regulate the dynamic of В-lymphocyte settling in peripheral organs. (da Silva et al., 2016).  (3) Pre-treatment modulates B lymphocytes and improves survival of melanoma-bearing animals.(Da Silva et al., 2020b) |
| B cells  Platelets | BUF, B(-) | (1) Increased B-cell proliferation from leukemic BALB/c mice (Hammarström et al., 1978; Shih et al., 2018) |
|  | OBN, C(+) | (1) Rise in membrane curvature leading to the generation of a procoagulant activity (Tomasiak et al., 2007) (due to inefficiently operating Na+/K(+)-ATPase and increased expression of phosphatidylserine) |
| Platelets | DGX, C(+) | (1) Activation of platelets in thrombosis-prone patients with heart failure and/or atrial fibrillation (Pettersen et al., 2002)  (2) Induced calcium mobilization (Chirinos et al., 2005)  (3) Increased levels of endothelial and platelet activation (Chirinos et al., 2005) |
|  |  |  |

C- cardenolide, B- bufadienolide, + with sugar residue, - without sugar residue.

References

Balzan, S., D’Urso, G., Nicolini, G., Forini, F., Pellegrino, M., and Montali, U. (2007). Erythrocyte sodium pump stimulation by ouabain and an endogenous ouabain-like factor. *Cell Biochem. Funct.* 25, 297–303.

Bauer, S., Groh, V., Wu, J., Steinle, A., Phillips, J. H., Lanier, L. L., et al. (1999). Activation of NK cells and T cells by NKG2D, a receptor for stress-inducible MICA. *Science* 285, 727–729. doi: 10.1126/SCIENCE.285.5428.727

Carvalho, D. C. M., Cavalcante-Silva, L. H. A., De A Lima, É., Galvão, J. G. F. M., De A Alves, A. K., Feijó, P. R. O., et al. (2019). Marinobufagenin Inhibits Neutrophil Migration and Proinflammatory Cytokines. *J. Immunol. Res.* 2019. doi: 10.1155/2019/1094520

Cavalcante‑Silva, L. H. A., Carvalho, D. C. M., de Almeida Lima, É., and Rodrigues‑Mascarenhas, S. (2021). Ouabain inhibits p38 activation in mice neutrophils. *Inflammopharmacology* 29, 1829–1833. doi: 10.1007/S10787-021-00882-Z

Chirinos, J. A., Castrellon, A., Zambrano, J. P., Jimenez, J. J., Jy, W., Horstman, L. L., et al. (2005). Digoxin use is associated with increased platelet and endothelial cell activation in patients with nonvalvular atrial fibrillation. *Hear. Rhythm* 2, 525–529. doi: 10.1016/J.HRTHM.2005.01.016

Da Silva, J. M. C., Azevedo, A. D. N., Barbosa, R. P. D. S., Teixeira, M. P., Vianna, T. A. G., Fittipaldi, J., et al. (2019). Ouabain Decreases Regulatory T Cell Number in Mice by Reducing IL-2 Secretion. *Neuroimmunomodulation* 26, 188–197. doi: 10.1159/000501720

Da Silva, J. M. C., Campos, M. L. A., Teixeira, M. P., da Silva Faustino, R., Aleixo, R. C., Cavalcante, F. J. P., et al. (2020a). Ouabain pre-treatment modulates B and T lymphocytes and improves survival of melanoma-bearing animals. *Int. Immunopharmacol.* 86. doi: 10.1016/J.INTIMP.2020.106772

Da Silva, J. M. C., Campos, M. L. A., Teixeira, M. P., da Silva Faustino, R., Aleixo, R. C., Cavalcante, F. J. P., et al. (2020b). Ouabain pre-treatment modulates B and T lymphocytes and improves survival of melanoma-bearing animals. *Int. Immunopharmacol.* 86, 106772.

da Silva, J. M. C., das Neves Azevedo, A., dos Santos Barbosa, R. P., Vianna, T. A. G., Fittipaldi, J., Teixeira, M. P., et al. (2016). Dynamics of murine B lymphocytes is modulated by in vivo treatment with steroid ouabain. *Immunobiology* 221, 368–376.

De Paiva, L. S., Costa, K. M. da, Canto, F. B. do, Cabral, V. R., Fucs, R., Nobrega, A., et al. (2011). Modulation of mature B cells in mice following treatment with ouabain. *Immunobiology* 216, 1038–1043. doi: 10.1016/J.IMBIO.2011.03.002

de Paiva, L. S., da Costa, K. M., do Canto, F. B., Cabral, V. R., Fucs, R., Nobrega, A., et al. (2011). Modulation of mature B cells in mice following treatment with ouabain. *Immunobiology* 216, 1038–1043.

Fedorova, O. V, Fadeev, A. V, Grigorova, Y. N., Marshall, C. A., Zernetkina, V., Kolodkin, N. I., et al. (2019). Cardiotonic Steroids Induce Vascular Fibrosis Via Pressure-Independent Mechanism in NaCl-Loaded Diabetic Rats. *J. Cardiovasc. Pharmacol.* 74, 436.

Fu, R., Yu, F., Wu, W., Liu, J., Li, J., Guo, F., et al. (2021). Bufalin enhances the killing efficacy of NK cells against hepatocellular carcinoma by inhibiting MICA shedding. *Int. Immunopharmacol.* 101. doi: 10.1016/J.INTIMP.2021.108195

Gao, Y., Xu, Z., Li, X., Liu, Z., Li, W., Kang, Y., et al. (2022). Resibufogenin, one of bufadienolides in toad venom, suppresses LPS-induced inflammation via inhibiting NF-κB and AP-1 pathways. *Int. Immunopharmacol.* 113, 109312.

Hammarström, L., Smith, C. I. E., and Persson, U. (1978). Functional Characterization of Lanatoside‐C‐Responsive Cells. *Scand. J. Immunol.* 8, 263–271.

Huang, Y., Yang, G., Fei, J., Wu, Y., and Yan, J. (2020). Bufotalin ameliorates experimental Sjögren’s syndrome development by inhibiting Th17 generation. *Naunyn. Schmiedebergs. Arch. Pharmacol.* 393, 1977–1985. doi: 10.1007/S00210-020-01817-1

Huh, J. R., Leung, M. W. L., Huang, P., Ryan, D. A., Krout, M. R., Malapaka, R. R. V., et al. (2011). Digoxin and its derivatives suppress TH17 cell differentiation by antagonizing RORγt activity. *Nature* 472, 486–490. doi: 10.1038/NATURE09978

Jacob, P. L., Leite, J. A., Alves, A. K. A., Rodrigues, Y. K. S., Amorim, F. M., Néris, P. L. N., et al. (2013). Immunomodulatory activity of ouabain in Leishmania leishmania amazonensis-infected Swiss mice. *Parasitol. Res.* 112, 1313–1321. doi: 10.1007/S00436-012-3146-9

Karaś, K., Sałkowska, A., Walczak-Drzewiecka, A., Ryba, K., Dastych, J., Bachorz, R. A., et al. (2018). The cardenolides strophanthidin, digoxigenin and dihydroouabain act as activators of the human RORγ/RORγT receptors. *Toxicol. Lett.* 295, 314–324. doi: 10.1016/J.TOXLET.2018.07.002

Khalaf, F. K., Dube, P., Kleinhenz, A. L., Malhotra, D., Gohara, A., Drummond, C. A., et al. (2019). Proinflammatory effects of cardiotonic steroids mediated by NKA α-1 (Na+/K+-ATPase α-1)/Src complex in renal epithelial cells and immune cells. *Hypertension* 74, 73–82.

Lee, J., Baek, S., Lee, J., Lee, J., Lee, D. G., Park, M. K., et al. (2015). Digoxin ameliorates autoimmune arthritis via suppression of Th17 differentiation. *Int. Immunopharmacol.* 26, 103–111. doi: 10.1016/J.INTIMP.2015.03.017

Li, X., Zheng, J., Chen, S., Meng, F., Ning, J., and Sun, S. (2020). Oleandrin Induces Immunogenic Cell Death Via the PERK/elF2α/ATF4/CHOP Pathway in Breast Cancer.

Manna, S. K., Sreenivasan, Y., and Sarkar, A. (2006). Cardiac glycoside inhibits IL-8-induced biological responses by downregulating IL-8 receptors through altering membrane fluidity. *J. Cell. Physiol.* 207, 195–207. doi: 10.1002/JCP.20555

Maxwell, K. D., Chuang, J., Chaudhry, M., Nie, Y., Bai, F., Sodhi, K., et al. (2021). The potential role of Na-K-ATPase and its signaling in the development of anemia in chronic kidney disease. *Am. J. Physiol. Physiol.* 320, F234–F242.

Nawaz, A., Aminuddin, A., Kado, T., Takikawa, A., Yamamoto, S., Tsuneyama, K., et al. (2017). CD206+ M2-like macrophages regulate systemic glucose metabolism by inhibiting proliferation of adipocyte progenitors. *Nat. Commun.* 8, 286.

Pettersen, E., Hagberg, I., Lyberg, T., and Gjesdal, K. (2002). Do cardiac glycosides affect platelet function? A flow cytometric study in healthy volunteers. *Eur. J. Clin. Pharmacol.* 58, 181–186. doi: 10.1007/S00228-002-0451-8

Ray, E., and Samanta, A. K. (1997). RECEPTOR-MEDIATED ENDOCYTOSIS OF IL-8: A FLUORESCENT MICROSCOPIC EVIDENCE AND IMPLICATION OF THE PROCESS IN LIGAND-INDUCED BIOLOGICAL RESPONSE IN HUMAN NEUTROPHILS. *Cytokine* 9, 587–596. doi: 10.1006/CYTO.1997.0206

Rodrigues-Mascarenhas, S., Dos Santos, N. F., and Rumjanek, V. M. (2006). Synergistic effect between ouabain and glucocorticoids for the induction of thymic atrophy. *Biosci. Rep.* 26, 159–169.

Shih, Y.-L., Chou, J.-S., Chen, Y.-L., Hsueh, S.-C., Chung, H.-Y., Lee, M.-H., et al. (2018). Bufalin enhances immune responses in leukemic mice through enhancing phagocytosis of macrophage in vivo. *In Vivo (Brooklyn).* 32, 1129–1136.

Smolarczyk, R., Cichoń, T., Pilny, E., Jarosz-Biej, M., Poczkaj, A., Kułach, N., et al. (2018). Combination of anti-vascular agent-DMXAA and HIF-1α inhibitor-digoxin inhibits the growth of melanoma tumors. *Sci. Rep.* 8, 7355.

Tomasiak, M., Stelmach, H., Rusak, T., Ciborowski, M., and Radziwon, P. (2007). The involvement of Na+/K(+)-ATPase in the development of platelet procoagulant response. *Acta Biochim. Pol.* 54, 625–639. doi: 10.18388/abp.2007_3236

Vieira, L., Saldanha, A. A., Moraes, A. M., Oliveira, F. M. de, Lopes, D. O., Barbosa, L. A. de O., et al. (2018). 21‑Benzylidene digoxin, a novel digoxin hemi-synthetic derivative, presents an anti-inflammatory activity through inhibition of edema, tumour necrosis factor alpha production, inducible nitric oxide synthase expression and leucocyte migration. *Int. Immunopharmacol.* 65, 174–181. doi: 10.1016/J.INTIMP.2018.10.010

Wu, S. C., Fu, B. D., Shen, H. Q., Yi, P. F., Zhang, L. Y., Lv, S., et al. (2015). Telocinobufagin enhances the Th1 immune response and protects against Salmonella typhimurium infection. *Int. Immunopharmacol.* 25, 353–362. doi: 10.1016/J.INTIMP.2015.02.005

Xiang, Y., Chen, L., Li, L., and Huang, Y. (2019). Restoration and enhancement of immunogenic cell death of cisplatin by coadministration with digoxin and conjugation to HPMA copolymer. *ACS Appl. Mater. Interfaces* 12, 1606–1616.

Yuan, B., He, J., Kisoh, K., Hayashi, H., Tanaka, S., Si, N., et al. (2016). Effects of active bufadienolide compounds on human cancer cells and CD4+CD25+Foxp3+ regulatory T cells in mitogen-activated human peripheral blood mononuclear cells. *Oncol. Rep.* 36, 1377–1384. doi: 10.3892/OR.2016.4946

Zhakeer, Z., Hadeer, M., Tuerxun, Z., and Tuerxun, K. (2017). Bufalin Inhibits the Inflammatory Effects in Asthmatic Mice through the Suppression of Nuclear Factor-Kappa B Activity. *Pharmacology* 99, 179–187. doi: 10.1159/000450754
